# Systemic Immunomodulatory Treatments for Atopic Dermatitis: Living Systematic Review and Network Meta-Analysis Update

**DOI:** 10.1001/jamadermatol.2024.2192

**Published:** 2024-07-17

**Authors:** Aaron M. Drucker, Megan Lam, David Prieto-Merino, Rayka Malek, Alexandra G. Ellis, Zenas Z. N. Yiu, Bram Rochwerg, Sonya Di Giorgio, Bernd W. M. Arents, Tanya Mohan, Tim Burton, Phyllis I. Spuls, Jochen Schmitt, Carsten Flohr

**Affiliations:** 1 Division of Dermatology, Department of Medicine, University of Toronto, Toronto, Ontario, Canada; 2 Department of Medicine and Women’s College Research Institute, Women’s College Hospital, Toronto, Ontario, Canada; 3 Faculty of Medicine, Universidad de Alcalá, Alcalá de Henares, Spain; 4 School of Life Course and Population Health Sciences, King’s College London, London, United Kingdom; 5 School of Public Health, Brown University, Providence, Rhode Island; 6 Division of Musculoskeletal and Dermatological Sciences, Faculty of Biology, Medicine and Health, The University of Manchester, Manchester, United Kingdom.; 7 Dermatology Centre, Manchester Academic Health Science Centre, Northern Care Alliance NHS Foundation Trust, Manchester, United Kingdom.; 8 Departments of Medicine and Health Research Methods, Evidence and Impact, McMaster University, Hamilton, Ontario, Canada; 9 Libraries & Collections, King’s College London, London, United Kingdom; 10 Dutch Association for People with Atopic Dermatitis, Nijkerk, the Netherlands; 11 Patient Representative (independent), Toronto, Ontario, Canada; 12 Patient Representative (independent), Nottingham, United Kingdom; 13 Department of Dermatology, Amsterdam Public Health/Infection and Immunology, Amsterdam, the Netherlands; 14 Center for Evidence-Based Healthcare, Faculty of Medicine Carl Gustav Carus, Technische Universität Dresden, Dresden, Germany; 15 Paediatric & Population-Based Dermatology Research, St John’s Institute of Dermatology, King’s College London and Guy’s and St Thomas’ NHS Foundation Trust, London, United Kingdom

## Abstract

**Importance:**

There are multiple approved systemic treatments for atopic dermatitis. Lebrikizumab is a newly licensed biologic medication that has been compared to placebo in clinical trials but not to other systemic treatments.

**Objective:**

To compare reported measures of efficacy and safety of lebrikizumab to other systemic treatments for atopic dermatitis in a living systematic review and network meta-analysis.

**Data Sources:**

The Cochrane Central Register of Controlled Trials, MEDLINE, Embase, the Latin American and Caribbean Health Science Information database, the Global Resource of Eczema Trials database, and trial registries were searched from inception through November 3, 2023.

**Study Selection:**

Randomized clinical trials evaluating 8 or more weeks of treatment with systemic immunomodulatory medications for moderate to severe atopic dermatitis. Titles, abstracts, and full texts were screened in duplicate.

**Data Extraction and Synthesis:**

Data were abstracted in duplicate and random-effects bayesian network meta-analyses were performed. Minimal important differences were used to define important differences between medications. Certainty of evidence was assessed using the GRADE approach (Grading of Recommendations Assessment, Development and Evaluation). The updated analysis was completed from December 13, 2023, to February 20, 2024.

**Main Outcome Measures:**

Efficacy outcomes were the Eczema Area and Severity Index (EASI), the Patient Oriented Eczema Measure (POEM) Dermatology Life Quality Index (DLQI), and Peak Pruritus Numeric Rating Scales (PP-NRS) and were compared using mean difference (MD) with 95% credible intervals (CrI). Safety outcomes were serious adverse events and withdrawal due to adverse events. Other outcomes included the proportion of participants with 50%, 75%, and 90% improvement in EASI (EASI-50, -75, -90) and the proportion with success on the Investigator Global Assessment compared using odds ratios with 95% CrI.

**Results:**

The study sample included 97 eligible trials, with a total of 24 679 patients. Lebrikizumab was associated with no important difference in change in EASI (MD, −2.0; 95% CrI, −4.5 to 0.3; moderate certainty), POEM (MD, −1.1; 95% CrI −2.5 to 0.2; moderate certainty), DLQI (MD, −0.2; 95% CrI −2.1 to 1.6; moderate certainty), or PP-NRS (MD, 0.1; 95% CrI −0.4, 0.6; high certainty) compared to dupilumab among adults with atopic dermatitis who were treated for up to 16 weeks. Dupilumab was associated with higher odds of efficacy in binary outcomes compared with lebrikizumab. The relative efficacy of other approved systemic medications was similar to that found by previous updates of this living study, with high-dose upadacitinib and abrocitinib demonstrating numerically highest relative efficacy. For safety outcomes, low event rates limited useful comparisons.

**Conclusions and Relevance:**

In this living systematic review and network meta-analysis, lebrikizumab was similarly effective to dupilumab for the short-term treatment of atopic dermatitis in adults. Clinicians and patients can use these comparative data to inform treatment decisions.

## Key Points

**Question:**

What is the relative efficacy and harm of lebrikizumab, the most recently approved biologic medication for atopic dermatitis compared with other systemic treatments?

**Findings:**

This systematic review and meta-analysis including 97 studies and 24 679 patients found moderate-certainty evidence that lebrikizumab is similarly effective to dupilumab for improving signs, symptoms, and quality of life for adults with atopic dermatitis after 16 weeks of treatment; however, a higher proportion of participants achieved treatment success with dupilumab according to binary outcomes.

**Meaning:**

These findings indicate that lebrikizumab, a new biologic medication, has comparable efficacy to dupilumab for the treatment of atopic dermatitis in adults.

## Teaser

This update of a living systematic review and network meta-analysis compares the efficacy and safety of systemic treatments for atopic dermatitis, including lebrikizumab.

## Introduction

Multiple systemic treatments have been approved since 2017 to treat atopic dermatitis. We are conducting a living systematic review and network meta-analysis (NMA) to enable comparisons between systemic medications, most of which have not been compared in head-to-head trials.^1,2,3,4^ Lebrikizumab is a monoclonal antibody targeting interleukin-13 that was approved by the European Medicines Agency in November 2023 to treat moderate to severe atopic dermatitis and is under review in other jurisdictions. Lebrikizumab has only been evaluated in placebo-controlled trials, so direct comparisons with other approved treatments cannot be made using existing clinical trials alone. This update to our living systematic review and NMA compares the efficacy and safety of systemic immunomodulatory treatments for atopic dermatitis, including lebrikizumab.

## Methods

This living systematic review and NMA is registered in PROSPERO (CRD42018088112) and has previously published detailed methods in a study protocol and prior updates with results.^1,2,3,4^ Given its design, this study does not require research ethics review or informed consent. We followed the Preferred Reporting Items for Systematic Reviews and Meta-analyses (PRISMA) reporting guideline for network meta-analysis.^5^

Our current search strategy is included as supplemental material in a recent publication.^4^ We search the Cochrane Central Register of Controlled Trials, MEDLINE, Embase, Latin American and Caribbean Health Science Information database, Global Resource of Eczema Trials database, ClinicalTrials.gov, and the World Health Organization’s International Clinical Trials Registry Platform every 4 months; for this update, we included trials from inception through November 3, 2023. We also publish ongoing updates on our website (http://www.eczematherapies.com/research).

In brief, this review includes randomized clinical trials evaluating systemic immunomodulatory treatments against any comparator, including placebo, given for 8 weeks or longer duration for atopic dermatitis (eMethods in Supplement 2). We included participants of any age with moderate to severe atopic dermatitis.

Abstract and full-text screening, data abstraction (eMethods in Supplement 2), and risk of bias assessments (at the study level using the Cochrane Risk of Bias tool, which includes selective outcome reporting)^6^ were performed by 2 investigators independently in duplicate (for this update, A.M.D. and M.L.). Any title or abstract marked as relevant by a single reviewer was advanced to full-text screening. Discrepancies in full-text screening, data abstraction, or risk of bias assessment were resolved by discussion between the reviewers, with adjudication by a senior investigator (C.F.), if needed.

Our main efficacy outcomes were change in clinical signs, prioritizing the Eczema Area and Severity Index (EASI); change in symptoms, prioritizing the Patient Oriented Eczema Measure (POEM); change in itch, prioritizing peak pruritus numeric rating scales (PP-NRS); and change in quality of life, prioritizing the Dermatology Life Quality Instrument (DLQI). Details on data abstraction for these continuous outcomes have been published previously.^2^ We also assessed 4 binary efficacy outcomes: the proportion of patients with 50%, 75%, and 90% improvement in EASI (EASI-50, -75, -90) and success on the Investigator Global Assessment (IGA) scales, prioritizing a reduction of 2 points and score of 0 or 1. Safety outcomes included the proportion of participants experiencing serious adverse events and withdrawal due to adverse events.

We performed random-effects bayesian NMA for each outcome using the GeMTC package for R, version 1.0-2 (van Valkenhoef G). We generate network plots where each node represents a unique dosing regimen for each medication. The width of each line connecting 2 treatments (nodes) is proportional to the number of head-to-head trials for that comparison. We qualitatively describe the geometry of the networks.

For continuous outcomes, we calculated mean differences with 95% credible intervals (CrI). Where outcome domains are measured using varied scales in different trials, we calculated standardized mean differences. Given that, with the exception of NMAs, the relative efficacy of most treatments in the network is uncertain, we used noninformative prior distributions for continuous efficacy outcomes.^7^ For binary outcomes, we calculated odds ratios (ORs) and 95% CrI between each pair of nodes in the network. We used a normal prior distribution on the log ORs such that the 95% coverage included log(1/30) to log(30).^8^ We summarized treatment rankings using Surface Under the Cumulative Ranking.^9^

We conducted analyses separately for trials of adults vs children, but included trials done in combined populations of adults and adolescents in the adult analysis if most of the study sample was composed of adults. We conducted stratified analyses among trials that allow vs do-not-allow concomitant topical anti-inflammatory medications. We conducted sensitivity analyses including only trials with low risk of bias. We assessed network coherence using node-splitting to compare direct and indirect estimates. We used Gelman-Rubin-Brooks plots of the Gelman-Rubin shrink factor and visually inspected trace plots and posterior density distributions.

We assessed the certainty of evidence for continuous efficacy outcomes and safety outcomes using Grading of Recommendations Assessment, Development and Evaluation (GRADE) criteria for NMAs (eMethods in Supplement 2).^10,11^ For this update, we modified our assessments of precision for interpreting differences between medications for continuous outcomes using guidance to contextualize results based on the minimal important difference (MID) for each outcome measure.^12,13,14,15^ We defined “no important difference” as less than half of the MID; “a small important reduction” as half to just less than the MID; and “a large important reduction” as greater than or equal to the MID. We used these thresholds for GRADE imprecision ratings.

In this article, we present effect estimates, CrI, Surface Under the Cumulative Ranking values, and certainty assessments for approved doses of abrocitinib, baricitinib, dupilumab, lebrikizumab, tralokinumab, and upadacitinib, and placebo for EASI, POEM, PP-NRS, and DLQI; results for complete networks and other outcomes are available from the authors on request.

## Results

**Figure 1.**
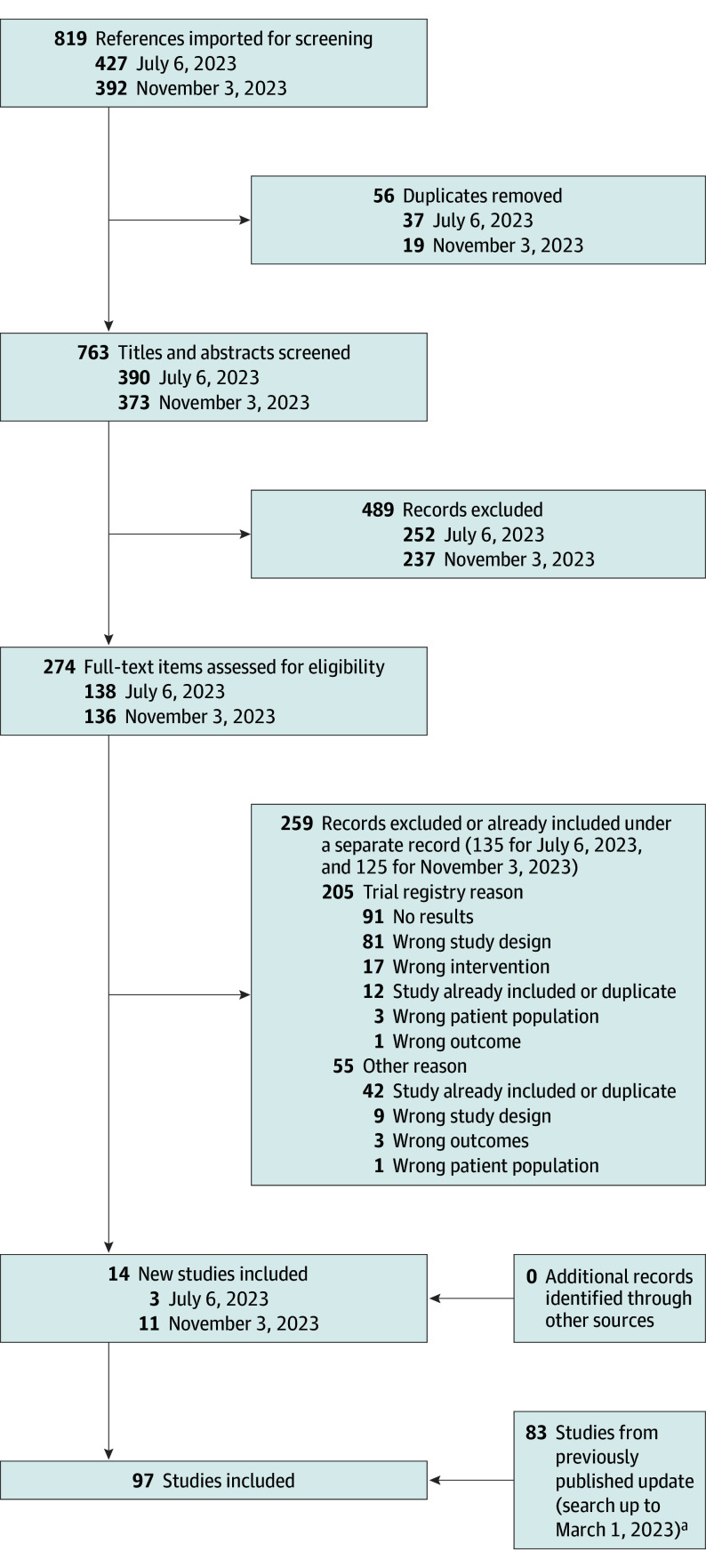
Study Screening and Selection Process for the Living Systematic Review of Systemic Immunomodulatory Treatments for Atopic Dermatitis, Including Results Up to the November 3, 2023 Search ^a^Most recent published PRISMA diagram was published by Drucker et al.^4^

As of November 3, 2023, there were 97 trials totaling 24 679 participants included in this systematic review and NMA (Figure 1).^16,17,18,19,20,21,22,23,24,25,26,27,28,29,30,31,32,33,34,35,36,37,38,39,40,41,42,43,44,45,46,47,48,49,50,51,52,53,54,55,56,57,58,59,60,61,62,63,64,65,66,67,68,69,70,71,72,73,74,75,76,77,78,79,80,81,82,83,84,85,86,87,88,89,90,91,92,93,94,95,96,97,98,99,100,101,102,103,104,105,106,107^ The characteristics of the trials currently included in the living systematic review, including extracted outcomes and risk of bias assessments, are included in Supplement 1 and can be accessed through our website (http://www.eczematherapies.com/research).

Network plots for all outcomes among adults reflect the predominance of placebo-controlled trials, with few direct connections between approved therapies (eFigure 1-8 in Supplement 2). Lebrikizumab was only connected to each network through placebo.

**Figure 2.**
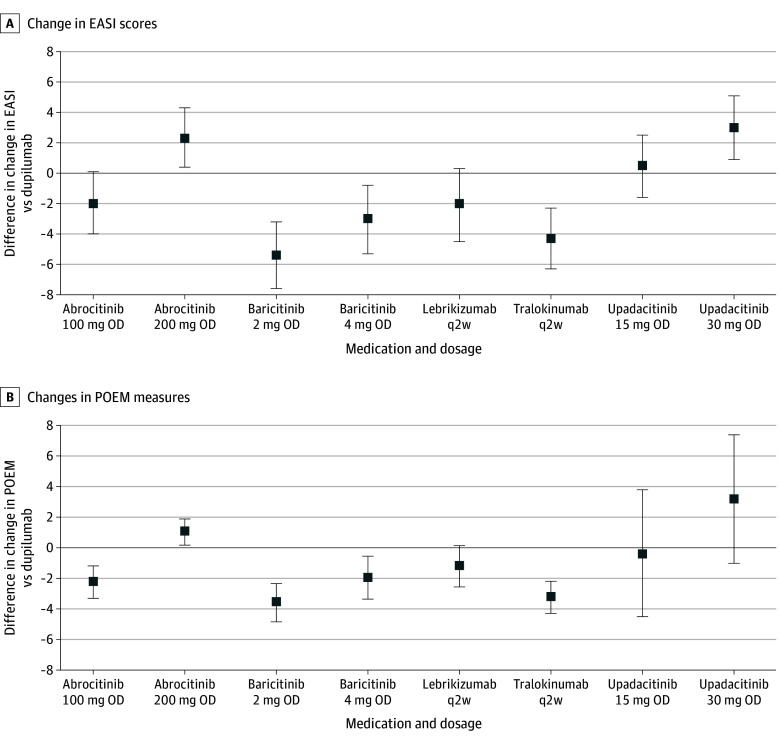
Network Meta-Analysis Results of Adults^a^ Treated for Between 8 and 16 Weeks for Change in Eczema Area And Severity Index (EASI) and Change in Patient Oriented Eczema Measure (POEM) Results are presented as mean difference (MD) with 95% credible intervals (CrI) for selected currently available targeted medications vs dupilumab. MDs greater than 0 indicate that the comparator is associated with more improvement than dupilumab. MDs less than 0 indicate that the comparator is associated with less improvement than dupilumab. Results for the complete networks are available on request. OD indicates once daily and q2w, every 2 weeks. ^a^Some studies included in the analyses of trials of adults include a minority proportion of adolescent (12-17 years old) participants.

**Figure 3.**
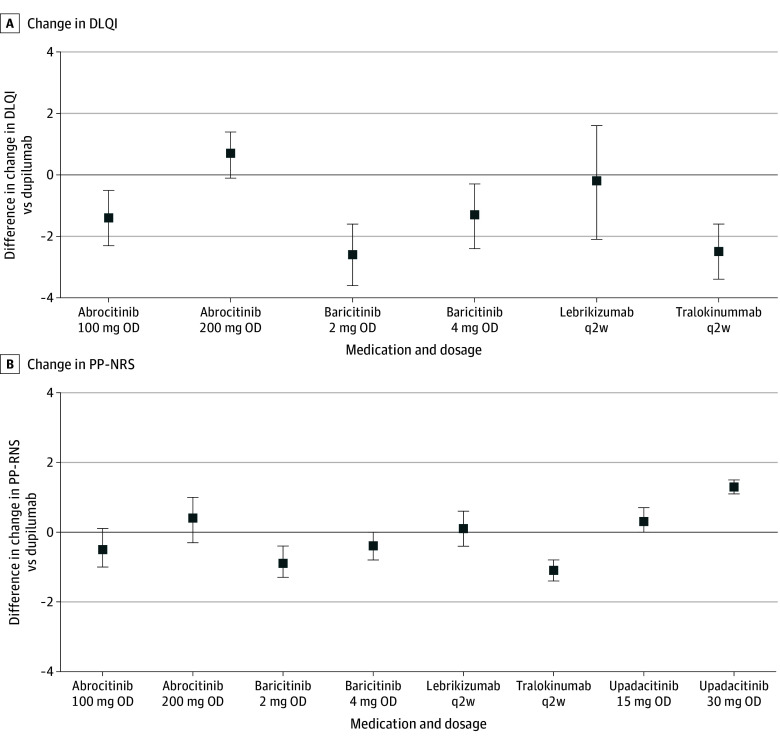
Network Meta-Analysis Results of Adults^a^ Treated for 8 to 16 Weeks for Changes in Dermatology Life Quality Index (DLQI) and in Peak Pruritus Numeric Rating Scales (PP-NRS) Results are presented as mean difference (MD) with 95% credible intervals (CrI) for selected currently available targeted medications vs dupilumab. MDs >0 indicate that the comparator is associated with more improvement than dupilumab. MDs <0 indicate that the comparator is associated with less improvement than dupilumab. Results for the complete networks are available upon request. OD indicates once daily and q2w, every 2 weeks. ^a^Some studies included in the analyses of trials of adults include a minority proportion of adolescent (12-17 years old) participants.

Compared to dupilumab (600 mg followed by 300 mg every 2 weeks), lebrikizumab (500 mg at weeks 0 and 2 followed by 250 mg every 2 weeks) was probably associated with no important difference in reductions in EASI (MD, −2.0; 95% CrI, −4.5 to 0.3; moderate certainty), POEM (MD, −1.1; 95% CrI, −2.5 to 0.2; moderate certainty), DLQI (MD −0.2; 95% CrI, −2.1 to 1.6; moderate certainty) scores, and was associated with no important difference in reduction in PP-NRS (MD 0.1; 95% CrI, −0.4 to 0.6; high certainty) scores in trials up to 16 weeks—positive numbers indicate more improvement with lebrikizumab (Figures 2 and 3; eTables 1-8 in Supplement 2). Dupilumab was associated with higher odds than lebrikizumab of achieving EASI-50 (OR, 1.4; 95% CrI, 1.0 to 2.0), EASI-75 (OR, 1.4; 95% CrI, 1.0 to 1.9), EASI-90 (OR, 1.5; 95% CrI, 1.1 to 2.2), and IGA success (OR, 1.3; 95% CrI, 0.9 to 1.9) (eTables 9-12 in Supplement 2). The relative efficacy of other approved systemic medications was similar to that found by previous NMA updates, with high-dose upadacitinib and abrocitinib demonstrating numerically highest relative efficacy. Results of efficacy analyses using standardized mean differences to compare newer to older medications (eg, methotrexate) and results of safety comparisons (serious adverse events, withdrawals due to adverse events) remain imprecise, with wide Crls.

There were no substantial differences in analyses separating trials that included topical anti-inflammatory treatments from those that did not. Excluding trials at high or uncertain risk of bias decreased the precision of estimates but did not change interpretation. Node splitting did not demonstrate significant incoherence. Networks limited to trials among children were associated with very imprecise estimates between treatments precluding clinically meaningful interpretations. Gelman-Rubin-Brooks plots, Gelman-Rubin shrink factor, trace plots, and posterior density distributions demonstrated good convergence features.

## Discussion

In this update of a living systematic review and NMA, there was moderate-certainty evidence that lebrikizumab has similar efficacy up to 16 weeks of treatment compared to dupilumab in improving signs, symptoms and quality of life associated with atopic dermatitis in adults. Dupilumab was associated with higher odds of achieving efficacy in binary outcomes than lebrikizumab.

The findings of this NMA are largely consistent with the findings of other recently published systematic reviews with NMA of systemic treatments for atopic dermatitis.^108,109^ Chu et al^108^ used a GRADE minimally contextualized approach to create categories of medications based on their relative efficacy. With that approach, they concluded that lebrikizumab, tralokinumab, and baricitinib are in a lower efficacy category than dupilumab when comparing improvements in EASI scores. In contrast, we used published MID values to contextualize the results as part of our GRADE assessment. This approach avoids creating arbitrary efficacy categories, and instead provides comparisons with GRADE certainty assessments between individual medications, taking into account the magnitude of the difference between them and the certainty of that estimate. The study by Chu et al^108^ included more trials in its networks than we did because it included results for children and adults in the same networks and had broader inclusion criteria. For example, that study^108^ did not exclude trials of shorter duration (whereas this study excluded those with <8 weeks of intervention) and it included some interventions that we did not consider to be systemic immunomodulators, such as phototherapy, histidine, and vitamin D. We believe these differences may make our review less prone to violations of the transitivity assumption, whereas Chu et al were able to make more comparisons within larger treatment networks. Chu et al also used different safety outcomes. They found statistical differences in overall adverse events between medications, whereas we did not detect important differences in withdrawals due to adverse events or serious adverse events, which are less common. The NMA by Silverberg et al^109^ was limited to studies of new targeted agents used without concomitant topical corticosteroids. Although that approach may improve transitivity compared to the more inclusive approach we used, we did not find any substantial differences with our secondary analyses limited to studies allowing vs not allowing concomitant topical anti-inflammatory therapy.

### Limitations

Trials including children with atopic dermatitis remain sparse, limiting the generalizability of our main findings for a disease that is more common among children. Atopic dermatitis is often chronic, necessitating long-term therapy. Because our study did not include long-term extension data from studies in which participants were rerandomized to different maintenance regimens, we were unable to draw conclusions about the relative efficacy of long-term dosing strategies. One of our objectives was to compare the safety of the medications under study, but our safety analyses were not clinically useful because of imprecise effect estimates. Furthermore, because broad adverse event categories (eg, serious adverse events and withdrawals) in trials for skin disease are nonspecific, with flares of the underlying disease often categorized as adverse events, safety data from randomized clinical trials can be difficult to interpret.^110^ While node splitting does not suggest incoherence in our networks, the limited number of head-to-head trials means those analyses were underpowered or, for most comparisons, not feasible. With multiple systemic treatments now available in routine clinical practice, trial populations may be changing over time. Specifically, baseline severity scores may be decreasing, which could affect the NMA transitivity assumption.^111^ Still, inclusion criteria are consistent across included trials over time, and it is unclear whether baseline differences would affect placebo and intervention groups differently. As more head-to-head trials are published for medications already approved, analyses can be done to assess the influence of these temporal trends.

## Conclusions

The findings of this update to a living systematic review and NMA support lebrikizumab as another effective biologic medication for treating atopic dermatitis. Although binary efficacy outcomes favored dupilumab, the differences in efficacy between dupilumab and lebrikizumab on continuous scales were small.

## Notes

Supplement 1. Dataset

Supplement 2. eMethods eFigure 1. Network plot for change in EASI eTable 1. League table for change in EASI eTable 2. GRADE certainty ratings for change in EASI eFigure 2. Network plot for change in POEM eTable 3. League table for change in POEM eTable 4. GRADE certainty ratings for change in POEM eFigure 3. Network plot for change in DLQI eTable 5. League table for change in DLQI eTable 6. GRADE certainty ratings for change in DLQI eFigure 4. Network plot for change in PP-NRS eTable 7. League table for change in PP-NRS eTable 8. GRADE certainty ratings for change in PP-NRS eFigure 5. Network plot for success achieving 50% improvement in EASI (EASI-50) eTable 9. League table for success achieving 50% improvement in EASI (EASI-50) eFigure 6. Network plot for success achieving 75% improvement in EASI (EASI-75) eTable 10. League table for success achieving 75% improvement in EASI (EASI-75) eFigure 7. Network plot for success achieving 90% improvement in EASI (EASI-90) eTable 11. League table for success achieving 90% improvement in EASI (EASI-90) eFigure 8. Network plot for achieving success on IGA eTable 12. League table for achieving success on IGA PRISMA NMA Checklist eReferences

Supplement 3. Data Sharing Statement
