# Supplementary material for: Systemic Immunomodulatory Treatments for Atopic Dermatitis: Living Systematic Review and Network Meta-Analysis Update
Source: JAMA Dermatol. 2024 Jul 17;160(9):936–44. doi: 10.1001/jamadermatol.2024.2192 (PMC11255974; doi:10.1001/jamadermatol.2024.2192)
Supplement: Supplement 2. — eMethods eFigure 1. Network plot for change in EASI eTable 1. League table for change in EASI eTable 2. GRADE certainty ratings for change in EASI eFigure 2. Network plot for change in POEM eTable 3. League table for change in POEM eTable 4. GRADE certainty ratings for change in POEM eFigure 3. Network plot for change in DLQI eTable 5. League table for change in DLQI eTable 6. GRADE certainty ratings for change in DLQI eFigure 4. Network plot for change in PP-NRS eTable 7. League table for change in PP-NRS eTable 8. GRADE certainty ratings for change in PP-NRS eFigure 5. Network plot for success achieving 50% improvement in EASI (EASI-50) eTable 9. League table for success achieving 50% improvement in EASI (EASI-50) eFigure 6. Network plot for success achieving 75% improvement in EASI (EASI-75) eTable 10. League table for success achieving 75% improvement in EASI (EASI-75) eFigure 7. Network plot for success achieving 90% improvement in EASI (EASI-90) eTable 11. League table for success achieving 90% improvement in EASI (EASI-90) eFigure 8. Network plot for achieving success on IGA eTable 12. League table for achieving success on IGA PRISMA NMA Checklist eReferences [file jamadermatol-e242192-s002.pdf]

Supplemental Online Content

Drucker AM, Lam M, Prieto-Merino D, et al. Systemic Immunomodulatory Treatments for Atopic Dermatitis: Living Systematic Review and Network Meta-Analysis Update. *JAMA Dermatol.* Published online July 17, 2024. doi:10.1001/jamadermatol.2024.2192

eMethods

eFigure 1. Network plot for change in EASI

eTable 1. League table for change in EASI

eTable 2. GRADE certainty ratings for change in EASI

eFigure 2. Network plot for change in POEM

eTable 3. League table for change in POEM

eTable 4. GRADE certainty ratings for change in POEM

eFigure 3. Network plot for change in DLQI

eTable 5. League table for change in DLQI

eTable 6. GRADE certainty ratings for change in DLQI

eFigure 4. Network plot for change in PP-NRS

eTable 7. League table for change in PP-NRS

eTable 8. GRADE certainty ratings for change in PP-NRS

eFigure 5. Network plot for success achieving 50% improvement in EASI (EASI-50)

eTable 9. League table for success achieving 50% improvement in EASI (EASI-50)

eFigure 6. Network plot for success achieving 75% improvement in EASI (EASI-75)

eTable 10. League table for success achieving 75% improvement in EASI (EASI-75)

eFigure 7. Network plot for success achieving 90% improvement in EASI (EASI-90)

eTable 11. League table for success achieving 90% improvement in EASI (EASI-90)

eFigure 8. Network plot for achieving success on IGA

eTable 12. League table for achieving success on IGA

eReferences

This supplemental material has been provided by the authors to give readers additional information about their work.

© 2024 American Medical Association. All rights reserved.

eMethods. *Inclusion criteria:* We include participants of any age with moderate-to-severe atopic dermatitis. We include parallel-group trials; for cross-over trials or trials where re-randomization occurs after an initial randomized period, we only include the initial randomized controlled trial period.

Data abstracted from included studies: We abstract data on studies' sites (single vs multicentre, since country vs international), age-related inclusion criteria, atopic dermatitis severity-related inclusion criteria, blinding, study duration, sponsor (industry vs other) and whether participants used concomitant topical anti-inflammatory therapy (categorized as yes or no; if only permitted as rescue therapy we categorize the study as not using concomitant topical therapy). For each trial arm, we extract the treatment regimen, the number of participants randomized and receiving treatment, the number of participants completing the initial randomized phase of the study, the mean participant age, the percentage of female participants and the mean atopic dermatitis severity, preferentially abstracting the Eczema Area and Severity Index (EASI) score. We extract data for our efficacy and safety outcomes, and we contact corresponding authors of studies published as peer-reviewed manuscripts to obtain missing outcomes data.

GRADE assessment: Each comparison is initially assigned a high certainty rating. Direct evidence can be rated down for risk of bias if the majority of evidence has high risk of bias and if there is evidence of important inconsistency based on a combination of visual inspection of forest plots, high I2 statistic (>40-50%) or Chi2 test (P<0.1). Indirect evidence can be rated down for the same reasons using the assessments for the lowest of the two direct estimates making up the dominant loop. The certainty assessment for the indirect evidence is then carried forward to the NMA assessment if there is no direct evidence, and the certainty assessment for the direct evidence is carried forward where there are no indirect connections. For comparisons where the direct evidence contributes a large majority of the weight to the network estimate (e.g., for comparisons between a given medication and placebo where the only trials for that medication are placebo-controlled), we also carry forward the certainty assessment of the direct estimate for the network estimate. For comparisons made up of both direct and indirect evidence, the higher of the direct or indirect rating are carried forward if there is no evidence of incoherence; if there is evidence of incoherence, we take the higher of the two estimates but rate down for incoherence. We then assess precision of the NMA estimate. For this update, we modified our assessments of precision used to interpret differences between medications for continuous outcomes using guidance to contextualize results based on the minimal important difference (MID) for each outcome measure.1-4 We defined "no important difference," as being within half of the MID; "a small important reduction," as lying between half of and just less than the MID; and "a large important reduction" as greater than or equal to the MID. We used these thresholds for GRADE imprecision ratings, rating down one level for estimates whose CrIs cross a half-MID unit threshold or two levels for CrIs that cross a full MID unit threshold. We created informative GRADE results statements incorporating effect estimates contextualized to the MID and final certainty ratings.5

© 2024 American Medical Association. All rights reserved.

eFigure 1. Network plot of studies included in the network meta-analysis of adults* treated between 8 and 16 weeks for change in Eczema Area and Severity Index (EASI).

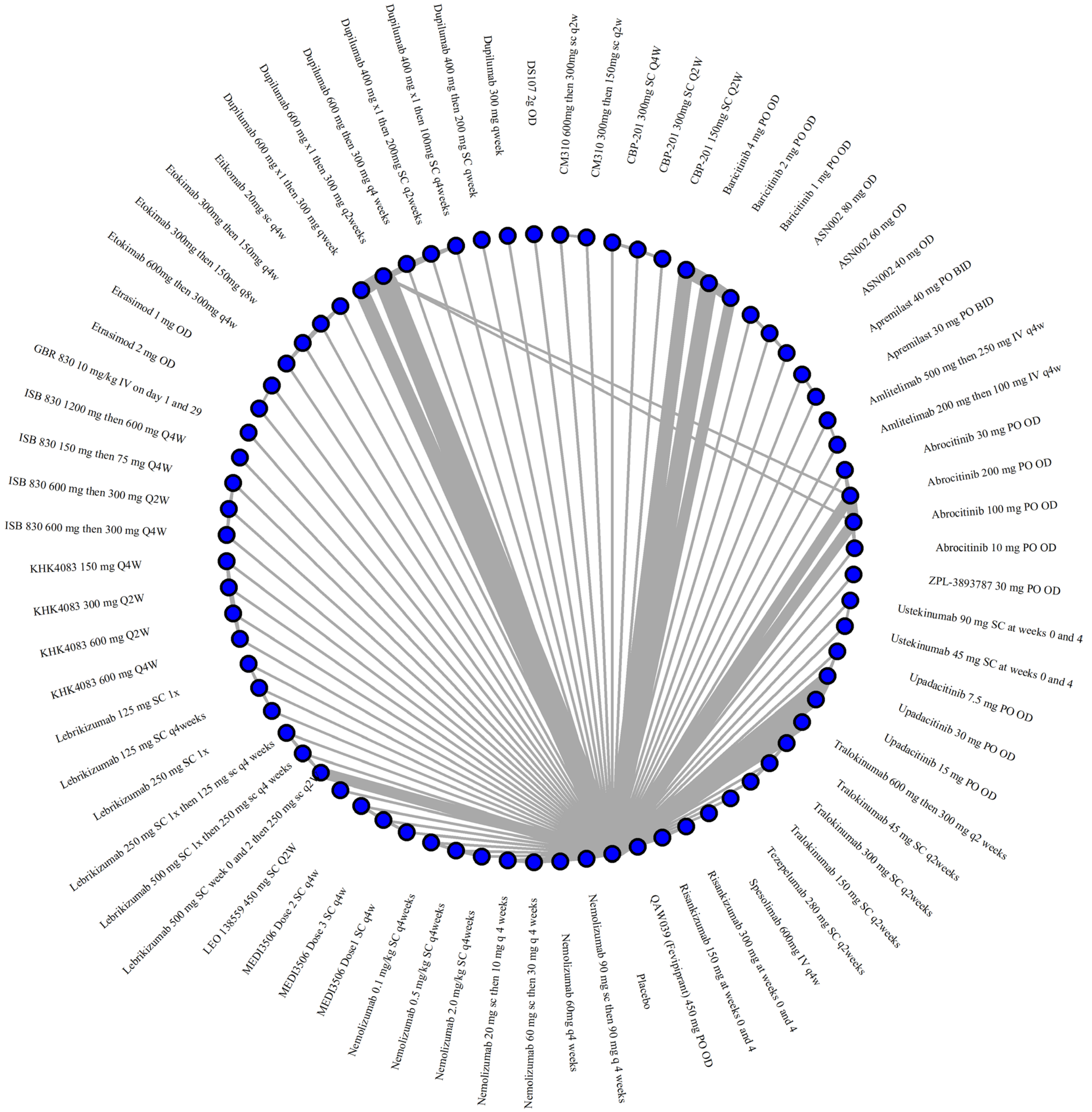

The width of each line connecting two treatments (nodes) is proportional to the number of head-to-head trials for that comparison.

OD: once daily; q1w: once weekly; q2w: every 2 weeks; q4w: every 4 weeks.

*Some studies included in the analyses of trials of adults include a minority proportion of adolescent (12-17 years old) participants

© 2024 American Medical Association. All rights reserved.

eTable 1. Relative effect estimates for change in EASI up to 16 weeks of treatment in adults for placebo and medications used in clinical practice. Results for other pairwise comparisons in the network can be requested from the authors. Results are presented as change in EASI (95% CrI). A positive effect estimate in a given cell favors the row-defining treatment. A negative effect estimate in a given cell favors the column-defining treatment. The bottom row contains the Surface Under the Cumulative Ranking (SUCRA) value for the column-defining treatment.

	Abro 100	Abro 200	Bari 2	Bari 4	Dupi	Lebri	Placebo	Tralo	Upa 15	Upa 30
Abro 100		-4.3 (-6.1, -2.6)	3.4 (0.9, 5.8)	1 (-1.6, 3.6)	-2 (-4, -0.1)	0 (-2.6, 2.7)	8.5 (6.7, 10.3)	2.3 (-0.1, 4.6)	-2.5 (-4.9, -0.1)	-5 (-7.4, -2.6)
Abro 200	4.3 (2.6, 6.1)		7.7 (5.2, 10.2)	5.3 (2.8, 7.9)	2.3 (0.4, 4.3)	4.4 (1.8, 7)	12.8 (11.1, 14.6)	6.6 (4.3, 8.9)	1.9 (-0.6, 4.2)	-0.7 (-3.1, 1.7)
Bari 2	-3.4 (-5.8, -0.9)	-7.7 (-10.2, -5.2)		-2.4 (-4.3, -0.5)	-5.4 (-7.6, -3.2)	-3.4 (-5.9, -0.7)	5.1 (3.4, 6.9)	-1.1 (-3.4, 1.2)	-5.9 (-8.2, -3.5)	-8.4 (-10.8, -6)
Bari 4	-1 (-3.6, 1.6)	-5.3 (-7.9, -2.8)	2.4 (0.5, 4.3)		-3 (-5.3, -0.8)	-0.9 (-3.6, 1.8)	7.5 (5.6, 9.4)	1.3 (-1.1, 3.7)	-3.5 (-6, -1)	-6 (-8.5, -3.6)
Dupi	2 (0.1, 4)	-2.3 (-4.3, -0.4)	5.4 (3.2, 7.6)	3 (0.8, 5.3)		2 (-0.3, 4.5)	10.5 (9.2, 11.9)	4.3 (2.3, 6.3)	-0.5 (-2.5, 1.6)	-3 (-5.1, -0.9)
Lebri	0 (-2.7, 2.6)	-4.4 (-7, -1.8)	3.4 (0.7, 5.9)	0.9 (-1.8, 3.6)	-2 (-4.5, 0.3)		8.5 (6.5, 10.4)	2.2 (-0.3, 4.7)	-2.5 (-5.1, 0)	-5.1 (-7.6, -2.6)
Placebo	-8.5 (-10.3, -6.7)	-12.8 (-14.6, -11.1)	-5.1 (-6.9, -3.4)	-7.5 (-9.4, -5.6)	-10.5 (-11.9, -9.2)	-8.5 (-10.4, -6.5)		-6.2 (-7.8, -4.7)	-11 (-12.6, -9.4)	-13.5 (-15.2, -11.9)
Tralo	-2.3 (-4.6, 0.1)	-6.6 (-8.9, -4.3)	1.1 (-1.2, 3.4)	-1.3 (-3.7, 1.1)	-4.3 (-6.3, -2.3)	-2.2 (-4.7, 0.3)	6.2 (4.7, 7.8)		-4.7 (-7, -2.5)	-7.3 (-9.5, -5.1)
Upa 15	2.5 (0.1, 4.9)	-1.9 (-4.2, 0.6)	5.9 (3.5, 8.2)	3.5 (1, 6)	0.5 (-1.6, 2.5)	2.5 (0, 5.1)	11 (9.4, 12.6)	4.7 (2.5, 7)		-2.5 (-4.1, -1)
Upa 30	5 (2.6, 7.4)	0.7 (-1.7, 3.1)	8.4 (6, 10.8)	6 (3.6, 8.5)	3 (0.9, 5.1)	5.1 (2.6, 7.6)	13.5 (11.9, 15.2)	7.3 (5.1, 9.5)	2.5 (1, 4.1)	
SUCRA	0.75	0.92	0.51	0.69	0.85	0.75	0.12	0.60	0.87	0.94

Abro 100/200: abrocitinib 100 mg/200 mg daily; bari 2/4: baricitinib 2mg/4 mg daily; dupi: dupilumab 600 mg then 300 mg every 2 weeks; lebri: lebrikizumab 500 mg at week 0 and 2 then 250 mg every 2 weeks; tralo: tralokinumab 600 mg then 300 mg every 2 weeks; upa 15/30: upadacitinib 15mg/30 mg daily.

© 2024 American Medical Association. All rights reserved.

eTable 2. GRADE certainty ratings for change in EASI up to 16 weeks of treatment in adults for placebo and medications used in clinical practice. Results are presented as change in EASI (95% CrI). Negative effect estimates favor the treatment listed in the intervention column; positive effect estimates favor the comparator.

Intervention	Comparator	Head-to-head trials, n	Direct estimate (95% CI); Certainty of Evidence*	Indirect estimate (95% CrI); Certainty of Evidence^	NMA Estimate (95% CrI); Certainty of Evidence
Abrocitinib 100 mg daily	Abrocitinib 200 mg daily	4	4.2 (3.0, 5.3); high	--	4.3 (2.6, 6.1); moderate[b]
Abrocitinib 200 mg daily is probably associated with a small important reduction in EASI scores vs Abrocitinib 100 mg daily.					
Abrocitinib 100 mg daily	Baricitinib 2 mg daily	0	--	-3.4 (-5.8, -0.9); high	-3.4 (-5.8, -0.9); moderate[b]
Abrocitinib 100 mg daily is probably associated with a large important reduction in EASI scores vs Baricitinib 2 mg daily.					
Abrocitinib 100 mg daily	Baricitinib 4 mg daily	0	--	-1 (-3.6, 1.6); high	-1 (-3.6, 1.6); moderate[b]
Abrocitinib 100 mg daily is probably associated with no important difference in reduction in EASI scores vs Baricitinib 4 mg daily.					
Abrocitinib 100 mg daily	Dupilumab	1	0.6 (-1, 2.1); high	2 (0.1, 4); high	2 (0.1, 4); moderate[b]
Abrocitinib 100 mg daily is probably associated with no important difference in reduction in EASI scores vs Dupilumab.					
Abrocitinib 100 mg daily	Lebrikizumab	0	--	0 (-2.7, 2.6); high	0 (-2.7, 2.6); high
Abrocitinib 100 mg daily is associated with no important difference in reduction in EASI scores vs Lebrikizumab.					
Abrocitinib 100 mg daily	Placebo	4	-7.9 (-9.3, -6.5); high	--	-8.5 (-10.3, -6.7); high
Abrocitinib 100 mg daily is associated with a large important reduction in EASI scores vs placebo.					
Abrocitinib 100 mg daily	Tralokinumab	0	--	2.3 (-4.6, 0.1); moderate[a]	-2.3 (-4.6, 0.1); low[a,b]
Abrocitinib 100 mg daily may be associated with no important difference in reduction in EASI scores vs Dupilumab.					
Abrocitinib 100 mg daily	Upadacitinib 15 mg daily	0	--	2.5 (0.1, 4.9); high	2.5 (0.1, 4.9); moderate[b]
Abrocitinib 100 mg daily is probably associated with no important difference in reduction in EASI scores vs Upadacitinib 15 mg daily.					
Abrocitinib 100 mg daily	Upadacitinib 30 mg daily	0	--	5 (2.6, 7.4); high	5 (2.6, 7.4); moderate[b]
Upadacitinib 30 mg daily is probably associated with a small important reduction in EASI scores vs Abrocitinib 100 mg daily.					
Abrocitinib 200 mg daily	Baricitinib 2 mg daily	0	--	-7.7 (-10.2, -5.2); high	-7.7 (-10.2, -5.2); moderate[b]
Abrocitinib 200 mg daily is probably associated with a large important reduction in EASI scores vs Baricitinib 2 mg daily.					
Abrocitinib 200 mg daily	Baricitinib 4 mg daily	0	--	-5.3 (-7.9, -2.8); high	-5.3 (-7.9, -2.8); moderate[b]
Abrocitinib 200 mg daily is probably associated with a small important reduction in EASI scores vs Baricitinib 4 mg daily.					
Abrocitinib 200 mg daily	Dupilumab	1	-3.0 (-4.5, -1.4); high	-1.8 (-4.4, 0.8); high	-2.3 (-4.3, -0.4); moderate[b]
Abrocitinib 200 mg daily is probably associated with no important difference in reduction in EASI scores vs Dupilumab.					
Abrocitinib 200 mg daily	Lebrikizumab	0	--	-4.4 (-7, -1.8); high	-4.4 (-7, -1.8); moderate[b]
Abrocitinib 200 mg daily is probably associated with a small important difference in reduction in EASI scores vs Lebrikizumab.					
Abrocitinib 200 mg daily	Placebo	4	-12.2 (-13.8, -10.6); high	--	-12.8 (-14.6, -11.1); high

© 2024 American Medical Association. All rights reserved.

Abrocitinib 200 mg daily is associated with a large important reduction in EASI scores vs placebo.					
Abrocitinib 200 mg daily	Tralokinumab	0	--	-6.6 (-8.9, -4.3); moderate[a]	-6.6 (-8.9, -4.3); low[a,b]
Abrocitinib 200 mg daily may be associated with a large important reduction in EASI scores vs Tralokinumab.					
Abrocitinib 200 mg daily	Upadacitinib 15 mg daily	0	--	-1.9 (-4.2, 0.6); high	-1.9 (-4.2, 0.6); moderate[b]
Abrocitinib 200 mg daily is probably associated with no important difference in reduction in EASI scores vs Upadacitinib 15 mg daily.					
Abrocitinib 200 mg daily	Upadacitinib 30 mg daily	0	--	0.7 (-1.7, 3.1); high	0.7 (-1.7, 3.1); high
Abrocitinib 200 mg daily is associated with no important difference in reduction in EASI scores vs Upadacitinib 30 mg daily.					
Baricitinib 2 mg daily	Baricitinib 4 mg daily	5	2.41 (-0.78, 4.1); moderate[a]	--	2.4 (0.5, 4.3); low[a,b]
Baricitinib 2 mg daily may be associated with no important difference in reduction in EASI scores vs Baricitinib 4 mg daily.					
Baricitinib 2 mg daily	Dupilumab	0	--	5.4 (3.2, 7.6); high	5.4 (3.2, 7.6); moderate[b]
Dupilumab is probably associated with a small important reduction in EASI scores vs Baricitinib 2 mg daily.					
Baricitinib 2 mg daily	Lebrikizumab	0	--	3.4 (0.7, 5.9); high	3.4 (0.7, 5.9); moderate[b]
Lebrikizumab is probably associated with a small important reduction in EASI scores vs Baricitinib 2 mg daily.					
Baricitinib 2 mg daily	Placebo	5	-5.2 (-6.6, -3.7); high	--	-5.1 (-6.9, -3.4); moderate[b]
Baricitinib 2 mg daily is probably associated with a small important reduction in EASI scores vs placebo.					
Baricitinib 2 mg daily	Tralokinumab	0	--	1.1 (-1.2, 3.4); moderate[a]	1.1 (-1.2, 3.4); low[a,b]
Baricitinib 2 mg daily may be associated with no important difference in reduction in EASI scores vs Tralokinumab.					
Baricitinib 2 mg daily	Upadacitinib 15 mg daily	0	--	5.9 (3.5, 8.2); high	5.9 (3.5, 8.2); moderate[b]
Upadacitinib 15 mg daily is probably associated with a small important reduction in EASI scores vs Baricitinib 2 mg daily.					
Baricitinib 2 mg daily	Upadacitinib 30 mg daily	0	--	8.4 (6, 10.8); high	8.4 (6, 10.8); moderate[b]
Upadacitinib 30 mg daily is probably associated with a large important reduction in EASI scores vs Baricitinib 2 mg daily.					
Baricitinib 4 mg daily	Dupilumab	0	--	3 (0.8, 5.3); high	3 (0.8, 5.3); moderate[b]
Baricitinib 4 mg daily is probably associated with no important difference in reduction in EASI scores vs Dupilumab.					
Baricitinib 4 mg daily	Lebrikizumab	0	--	0.9 (-1.8, 3.6); high	0.9 (-1.8, 3.6); moderate[b]
Baricitinib 4 mg daily is probably associated with no important difference in reduction in EASI scores vs Lebrikizumab.					
Baricitinib 4 mg daily	Placebo	4	-7.6 (-9.2, -6.0); high	--	-7.5 (-9.4, -5.6); moderate[b]
Baricitinib 4 mg daily is probably associated with a large important reduction in EASI scores vs placebo.					
Baricitinib 4 mg daily	Tralokinumab	0	--	-1.3 (-3.7, 1.1); moderate[a]	-1.3 (-3.7, 1.1); low[a,b]
Baricitinib 4 mg daily may be associated with no important difference in reduction in EASI scores vs Tralokinumab.					
Baricitinib 4 mg daily	Upadacitinib 15 mg daily	0	--	3.5 (1, 6); high	3.5 (1, 6); moderate[b]
Upadacitinib 15 mg daily is probably associated with a small important reduction in EASI scores vs Baricitinib 4 mg daily.					
Baricitinib 4 mg daily	Upadacitinib 30 mg daily	0	--	6 (3.6, 8.5); high	6 (3.6, 8.5); moderate[b]
Upadacitinib 30 mg daily is probably associated with a small important reduction in EASI scores vs Baricitinib 4 mg daily.					

© 2024 American Medical Association. All rights reserved.

Dupilumab	Lebrikizumab	0	--	-2 (-4.5, 0.3); high	-2 (-4.5, 0.3); moderate[b]
Dupilumab is probably associated with no important difference in reduction in EASI scores vs Lebrikizumab.					
Dupilumab	Placebo	7	-10.7 (-12.2, -9.1); high	--	-10.5 (-11.9, -9.2); high
Dupilumab is associated with a large important reduction in EASI scores vs placebo.					
Dupilumab	Tralokinumab	0	--	-4.3 (-6.3, -2.3); moderate[a]	-4.3 (-6.3, -2.3); low[a,b]
Dupilumab may be associated with a small important reduction in EASI scores vs Tralokinumab.					
Dupilumab	Upadacitinib 15 mg daily	0	--	0.5 (-1.6, 2.5); high	0.5 (-1.6, 2.5); high
Dupilumab is associated with no important difference in reduction in EASI scores vs Upadacitinib 15 mg daily.					
Dupilumab	Upadacitinib 30 mg daily	0	--	3 (0.9, 5.1); high	3 (0.9, 5.1); moderate[b]
Dupilumab is probably associated with no important difference in reduction in EASI scores vs Upadacitnib 30 mg daily.					
Lebrikizumab	Placebo	4	-8.6 (-10.11, -7.08); high	-8.5 (-10.4, -6.5); high	-8.5 (-10.4, -6.5); moderate[b]
Lebrikizumab is probably associated with a large important reduction in EASI scores vs Placebo.					
Lebrikizumab	Tralokinumab	0	--	-2.2 (-4.7, 0.3); moderate[a]	-2.2 (-4.7, 0.3); low[a,b]
Lebrikizumab may be associated with no important difference in reduction in EASI scores vs Tralokinumab.					
Lebrikizumab	Upadacitinib 15 mg daily	0	--	2.5 (0, 5.1); high	2.5 (0, 5.1); moderate[b]
Lebrikizumab is probably associated with no important difference in reduction in EASI scores vs Tralokinumab.					
Lebrikizumab	Upadacitinib 30 mg daily	0	--	5.1 (2.6, 7.6); high	5.1 (2.6, 7.6); moderate[b]
Upadacitinib 30 mg daily is probably associated with a small important reduction in EASI scores vs Lebrikizumab.					
Tralokinumab	Upadacitinib 15 mg daily	0	--	4.7 (2.5, 7); moderate[a]	4.7 (2.5, 7); low[a,b]
Upadacitinib 15 mg daily may be associated with a small important reduction in EASI scores vs Tralokinumab.					
Tralokinumab	Upadacitinib 30 mg daily	0	--	7.3 (5.1, 9.5); moderate[a]	7.3 (5.1, 9.5); low[a,b]
Upadacitinib 30 mg daily may be associated with a large important reduction in EASI scores vs Tralokinumab.					
Tralokinumab	Placebo	5	-6.2 (-8.3, -4.2); moderate[a]	--	-6.2 (-7.8, -4.7); low[a,b]
Tralokinumab may be associated with a small important reduction in EASI scores vs placebo.					
Upadacitinib 15 mg daily	Upadacitinib 30 mg daily	4	2.5 (0.9, 4.2); high	--	2.5 (1, 4.1); moderate[b]
Upadacitinib 15 mg daily is probably associated with no important difference in reduction in EASI scores vs Upadacitinib 30 mg daily.					
Upadacitinib 15 mg daily	Placebo	4	-11.0 (-13.0, -9.0); high	--	-11 (-12.6, -9.4); high
Upadacitinib 15 mg daily is associated with a large important reduction in EASI scores vs placebo.					
Upadacitinib 30 mg daily	Placebo	4	-13.6 (-15.1, -12.0); high	--	-13.5 (-15.2, -11.9); high
Upadacitinib 30 mg daily is associated with a large important reduction in EASI scores vs placebo.					

Dose of dupilumab and tralokinumab is 600 mg then 300 mg every 2 weeks.
Dose of lebrikizumab is 500 mg at weeks 0 and 2 then 250 mg every 2 weeks.

© 2024 American Medical Association. All rights reserved.

CI: Confidence interval. CrI: Credible interval.

The minimal important difference for EASI is 6.6.[2]

*Direct estimates, when available, are derived from random-effects frequentist pairwise meta-analysis.
^Indirect effects are taken from node splitting analyses when available; otherwise, the network effect is used.

a. Rated down for inconsistency
b. Rated down for imprecision

© 2024 American Medical Association. All rights reserved.

eFigure 2. Network plot of studies included in the network meta-analysis of adults* treated between 8 and 16 weeks for change in Patient Oriented Eczema Measure (POEM).

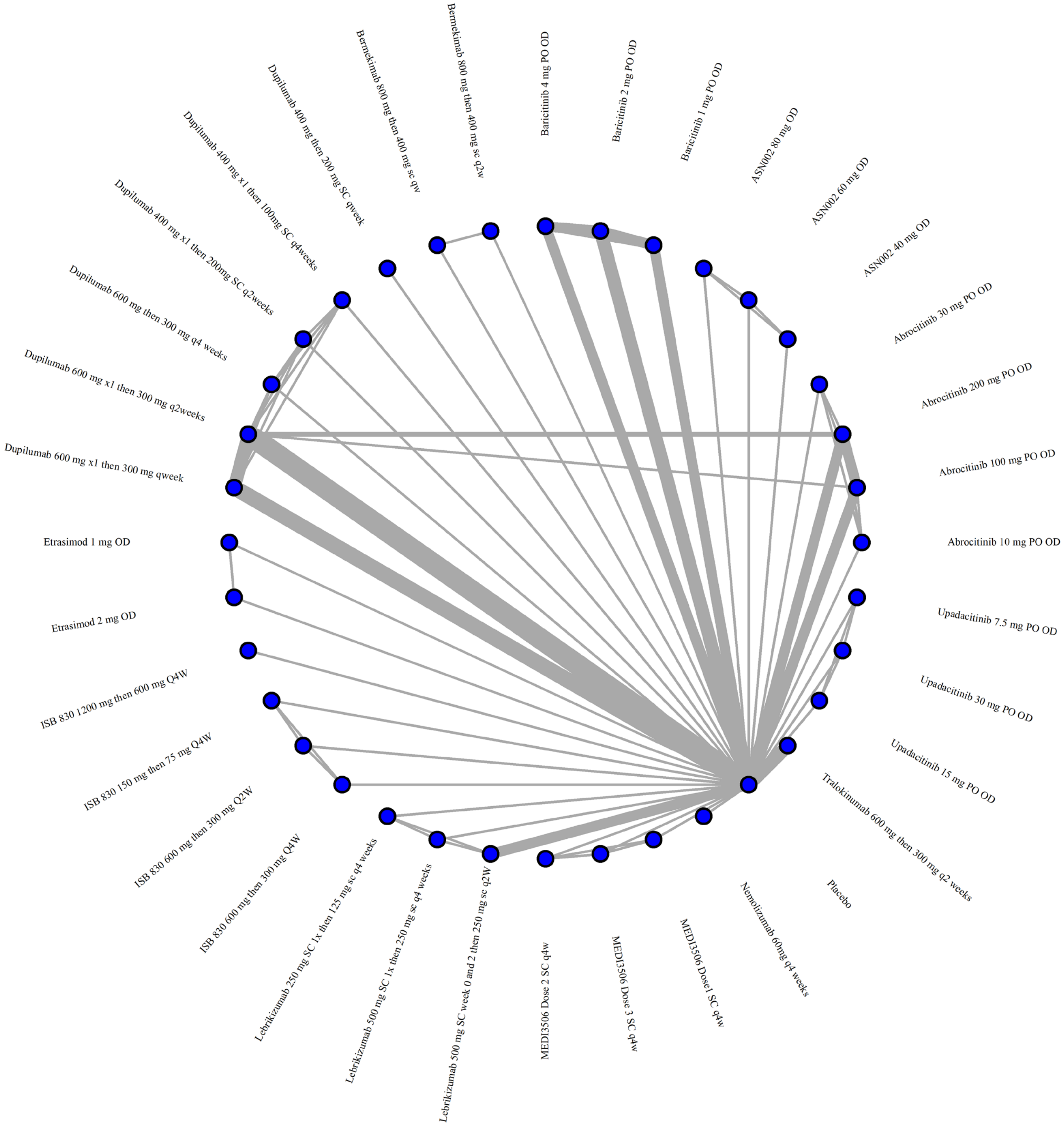

The width of each line connecting two treatments (nodes) is proportional to the number of head-to-head trials for that comparison.

OD: once daily; q1w: once weekly; q2w: every 2 weeks; q4w: every 4 weeks.

*Some studies included in the analyses of trials of adults include a minority proportion of adolescent (12-17 years old) participants

© 2024 American Medical Association. All rights reserved.

eTable 3. Relative effect estimates for change in POEM up to 16 weeks of treatment in adults for placebo and medications used in clinical practice. Results for other pairwise comparisons in the network can be requested from the authors. Results are presented as change in POEM (95% CrI). A positive effect estimate in a given cell favors the row-defining treatment. A negative effect estimate in a given cell favors the column-defining treatment. The bottom row contains the Surface Under the Cumulative Ranking (SUCRA) value for the column-defining treatment.

	Abro 100	Abro 200	Bari 2	Bari 4	Dupi	Lebri	Placebo	Tralo	Upa 15	Upa 30
Abro 100		-3.3 (-4.3, -2.4)	1.3 (-0.2, 2.8)	-0.3 (-1.9, 1.2)	-2.2 (-3.3, -1.2)	-1.1 (-2.6, 0.4)	5.1 (4.1, 6.1)	0.9 (-0.4, 2.2)	-1.9 (-6.2, 2.3)	-5.6 (-9.8, -1.3)
Abro 200	3.3 (2.4, 4.3)		4.6 (3.2, 6)	3 (1.5, 4.5)	1.1 (0.2, 1.9)	2.2 (0.7, 3.7)	8.4 (7.5, 9.3)	4.2 (3, 5.4)	1.4 (-2.9, 5.6)	-2.2 (-6.5, 2)
Bari 2	-1.3 (-2.8, 0.2)	-4.6 (-6, -3.2)		-1.7 (-2.8, -0.5)	-3.5 (-4.8, -2.3)	-2.5 (-4.1, -0.8)	3.8 (2.7, 4.9)	-0.4 (-1.8, 1)	-3.2 (-7.6, 1)	-6.9 (-11.2, -2.6)
Bari 4	0.3 (-1.2, 1.9)	-3 (-4.5, -1.5)	1.7 (0.5, 2.8)		-1.9 (-3.3, -0.5)	-0.8 (-2.5, 0.9)	5.4 (4.2, 6.6)	1.2 (-0.2, 2.7)	-1.5 (-5.9, 2.7)	-5.2 (-9.5, -0.9)
Dupi	2.2 (1.2, 3.3)	-1.1 (-1.9, -0.2)	3.5 (2.3, 4.8)	1.9 (0.5, 3.3)		1.1 (-0.2, 2.5)	7.3 (6.7, 8)	3.1 (2.1, 4.2)	0.3 (-3.9, 4.4)	-3.3 (-7.5, 0.9)
Lebri	1.1 (-0.4, 2.6)	-2.2 (-3.7, -0.7)	2.5 (0.8, 4.1)	0.8 (-0.9, 2.5)	-1.1 (-2.5, 0.2)		6.2 (5.1, 7.4)	2 (0.6, 3.5)	-0.7 (-5.1, 3.5)	-4.4 (-8.8, -0.2)
Placebo	-5.1 (-6.1, -4.1)	-8.4 (-9.3, -7.5)	-3.8 (-4.9, -2.7)	-5.4 (-6.6, -4.2)	-7.3 (-8, -6.7)	-6.2 (-7.4, -5.1)		-4.2 (-5, -3.4)	-7 (-11.2, -2.9)	-10.7 (-14.8, -6.5)
Tralo	-0.9 (-2.2, 0.4)	-4.2 (-5.4, -3)	0.4 (-1, 1.8)	-1.2 (-2.7, 0.2)	-3.1 (-4.2, -2.1)	-2 (-3.5, -0.6)	4.2 (3.4, 5)		-2.8 (-7.1, 1.3)	-6.4 (-10.7, -2.2)
Upa 15	1.9 (-2.3, 6.2)	-1.4 (-5.6, 2.9)	3.2 (-1, 7.6)	1.5 (-2.7, 5.9)	-0.3 (-4.4, 3.9)	0.7 (-3.5, 5.1)	7 (2.9, 11.2)	2.8 (-1.3, 7.1)		-3.7 (-7.7, 0.3)
Upa 30	5.6 (1.3, 9.8)	2.2 (-2, 6.5)	6.9 (2.6, 11.2)	5.2 (0.9, 9.5)	3.3 (-0.9, 7.5)	4.4 (0.2, 8.8)	10.7 (6.5, 14.8)	6.4 (2.2, 10.7)	3.7 (-0.3, 7.7)	
SUCRA	0.65	0.32	0.52	0.69	0.84	0.76	0.17	0.56	0.79	0.97

Abro 100/200: abrocitinib 100 mg/200 mg daily; bari 2/4: baricitinib 2mg/4 mg daily; dupi: dupilumab 600 mg then 300 mg every 2 weeks; lebri: lebrikizumab 500 mg at week 0 and 2 then 250 mg every 2 weeks; tralo: tralokinumab 600 mg then 300 mg every 2 weeks; upa 15/30: upadacitinib 15mg/30 mg daily.

© 2024 American Medical Association. All rights reserved.

eTable 4. GRADE certainty ratings for change in POEM up to 16 weeks of treatment in adults for placebo and medications used in clinical practice. Results are presented as change in EASI (95% CrI). Negative effect estimates favor the treatment listed in the intervention column; positive effect estimates favor the comparator.

Intervention	Comparator	Head-to-head trials, n	Direct estimate (95% CI); Certainty of Evidence*	Indirect estimate (95% CrI); Certainty of Evidence^	NMA Estimate (95% CrI); Certainty of Evidence
Abrocitinib 100 mg daily	Abrocitinib 200 mg daily	4	3.2 (2.3, 4.0); high	--	3.3 (2.4, 4.3); moderate[b]
Abrocitinib 200 mg daily is probably associated with a small important reduction in POEM scores vs Abrocitinib 100 mg daily.					
Abrocitinib 100 mg daily	Baricitinib 2 mg daily	0	--	-1.3 (-2.8, 0.2)	-1.3 (-2.8, 0.2); moderate[b]
Abrocitinib 100 mg daily is probably associated with no important difference in reduction in POEM scores vs Baricitinib 2 mg daily.					
Abrocitinib 100 mg daily	Baricitinib 4 mg daily	0	--	0.3 (-1.2, 1.9); high	0.3 (-1.2, 1.9); moderate[b]
Abrocitinib 100 mg daily is probably associated with no important difference in reduction in POEM scores vs Baricitinib 4 mg daily.					
Abrocitinib 100 mg daily	Dupilumab	1	1.6 (0.3, 2.9); high	2.5 (1.2, 4); high	2.2 (1.2, 3.3); moderate[b]
Dupilumab is probably associated with a small important reduction in POEM scores vs Abrocitinib 100 mg daily.					
Abrocitinib 100 mg daily	Lebrikizumab	0	--	1.1 (-0.4, 2.6); high	1.1 (-0.4, 2.6); moderate[b]
Abrocitinib 100 mg daily is probably associated with no important difference in reduction in POEM scores vs Lebrikizumab.					
Abrocitinib 100 mg daily	Placebo	4	-4.5 (-5.6, -3.4); high	--	-5.1 (-6.1, -4.1); high
Abrocitinib 100 mg daily is associated with a large important reduction in POEM scores vs placebo.					
Abrocitinib 100 mg daily	Tralokinumab	0	--	-0.9 (-2.2, 0.4); high	-0.9 (-2.2, 0.4); moderate[b]
Abrocitinib 100 mg daily is probably associated with no important difference in reduction in POEM scores vs Tralokinumab.					
Abrocitinib 100 mg daily	Upadacitinib 15 mg daily	0	--	1.9 (-2.3, 6.2); high	1.9 (-2.3, 6.2); low[c]
Upadacitinib 15 mg daily may be associated with a small important reduction in POEM scores vs Abrocitinib 100 mg daily.					
Abrocitinib 100 mg daily	Upadacitinib 30 mg daily	0	--	5.6 (1.3, 9.8); high	5.6 (1.3, 9.8); moderate[b]
Upadacitinib 30 mg daily is probably associated with a large important reduction in POEM scores vs Abrocitinib 100 mg daily.					
Abrocitinib 200 mg daily	Baricitinib 2 mg daily	0	--	-4.6 (-6, -3.2); high	-4.6 (-6, -3.2); moderate[b]
Abrocitinib 200 mg daily is probably associated with a large important reduction in POEM scores vs Baricitinib 2 mg daily.					
Abrocitinib 200 mg daily	Baricitinib 4 mg daily	0	--	-3 (-4.5, -1.5); high	-3 (-4.5, -1.5); moderate[b]
Abrocitinib 200 mg daily is probably associated with a small important reduction in POEM scores vs Baricitinib 4 mg daily.					
Abrocitinib 200 mg daily	Dupilumab	2	-1.5 (-2.2, -0.1); high	0.0 (-1.5, 1.5); high	-1.1 (-1.9, -0.2); moderate[b]
Abrocitinib 200 mg daily is probably associated with no important difference in reduction in POEM scores vs Dupilumab.					
Abrocitinib 200 mg daily	Lebrikizumab	0	--	-2.2 (-3.7, -0.7); high	-2.2 (-3.7, -0.7); moderate[b]
Abrocitinib 200 mg daily is probably associated with a small important reduction in POEM scores vs Lebrikizumab.					
Abrocitinib 200 mg daily	Placebo	4	-7.7 (-8.7, -6.7); high	-9.1 (-11.0, -7.7); high	-8.4 (-9.3, -7.5); high
Abrocitinib 200 mg daily is associated with a large important reduction in POEM scores vs placebo.					
Abrocitinib 200 mg daily	Tralokinumab	0	--	-4.2 (-5.4, -3); high	-4.2 (-5.4, -3); moderate[b]

© 2024 American Medical Association. All rights reserved.

Abrocitinib 200 mg daily is probably associated with a large important reduction in POEM scores vs Tralokinumab.					
Abrocitinib 200 mg daily	Upadacitinib 15 mg daily	0	--	-1.4 (-5.6, 2.9); high	-1.4 (-5.6, 2.9); low[c]
Abrocitinib 200 mg daily may be associated with no important difference in reduction in POEM scores vs Upadacitinib 15 mg daily.					
Abrocitinib 200 mg daily	Upadacitinib 30 mg daily	0	--	2.2 (-2, 6.5); high	2.2 (-2, 6.5); low[c]
Upadacitinib 30 mg daily may be associated with a small important reduction in POEM scores vs Abrocitinib 200 mg daily.					
Baricitinib 2 mg daily	Baricitinib 4 mg daily	4	1.7 (0.6, 2.8); high	--	1.7 (0.5, 2.8); moderate[b]
Baricitinib 4 mg daily is probably associated with a small important reduction in POEM scores vs Baricitinib 2 mg daily.					
Baricitinib 2 mg daily	Dupilumab	0	--	3.5 (2.3, 4.8); high	3.5 (2.3, 4.8); moderate[b]
Dupilumab is probably associated with a large important reduction in POEM scores vs Baricitinib 2 mg daily.					
Baricitinib 2 mg daily	Lebrikizumab	0	--	2.5 (0.8, 4.1); high	2.5 (0.8, 4.1); moderate[b]
Lebrikizumab is probably associated with a small important reduction in POEM scores vs Baricitinib 2 mg daily.					
Baricitinib 2 mg daily	Placebo	5	-3.8 (-4.8, -2.8); high	--	-3.8 (-4.9, -2.7); moderate[b]
Baricitinib 2 mg daily is probably associated with a large important reduction in POEM scores vs placebo.					
Baricitinib 2 mg daily	Tralokinumab	0	--	0.4 (-1, 1.8); high	0.4 (-1, 1.8); moderate[b]
Baricitinib 2 mg daily is probably associated with no important difference in reduction in POEM scores vs Tralokinumab.					
Baricitinib 2 mg daily	Upadacitinib 15 mg daily	0	--	3.2 (-1, 7.6); high	3.2 (-1, 7.6); low[c]
Upadacitinib 15 mg daily may be associated with a small important reduction in POEM scores vs Baricitinib 2 mg daily.					
Baricitinib 2 mg daily	Upadacitinib 30 mg daily	0	--	6.9 (2.6, 11.2); high	6.9 (2.6, 11.2); moderate[b]
Upadacitinib 30 mg daily is probably associated with a large important reduction in POEM scores vs Baricitinib 2 mg daily.					
Baricitinib 4 mg daily	Dupilumab	0	--	1.9 (0.5, 3.3); high	1.9 (0.5, 3.3); moderate[b]
Dupilumab is probably associated with a small important reduction in POEM scores vs Baricitinib 4 mg daily.					
Baricitinib 4 mg daily	Lebrikizumab	0	--	0.8 (-0.9, 2.5); high	0.8 (-0.9, 2.5); moderate[b]
Baricitinib 4 mg daily is probably associated with no important difference in reduction in POEM scores vs Lebrikizumab.					
Baricitinib 4 mg daily	Placebo	4	-5.4 (-6.5, -4.2); high	--	-5.4 (-6.6, -4.2); high
Baricitinib 4 mg daily is associated with a large important reduction in POEM scores vs placebo.					
Baricitinib 4 mg daily	Tralokinumab	0	--	-1.2 (-2.7, 0.2); high	-1.2 (-2.7, 0.2); moderate[b]
Baricitinib 4 mg daily is probably associated with no important difference in reduction in POEM scores vs Tralokinumab.					
Baricitinib 4 mg daily	Upadacitinib 15 mg daily	0	--	1.5 (-2.7, 5.9); high	1.5 (-2.7, 5.9); low[c]
Baricitinib 4 mg daily may be associated with no important difference in reduction in POEM scores vs Upadacitinib 15 mg daily.					
Baricitinib 4 mg daily	Upadacitinib 30 mg daily	0	--	5.2 (0.9, 9.5); high	5.2 (0.9, 9.5); low[c]
Upadacitinib 30 mg daily may be associated with a large important reduction in POEM scores vs Baricitinib 4 mg daily.					
Dupilumab	Lebrikizumab	0	--	-1.1 (-2.5, 0.2); high	-1.1 (-2.5, 0.2); moderate[b]
Dupilumab is probably associated with no important difference in reduction in POEM scores vs Lebrikizumab.					
Dupilumab	Placebo	8	-7.4 (-8.2, -6.7); high	-7.3 (-8, -6.7); high	-7.3 (-8, -6.7); high
Dupilumab is associated with a large important reduction in POEM scores vs placebo.					
Dupilumab	Tralokinumab	0	--	-3.1 (-4.2, -2.1); high	-3.1 (-4.2, -2.1); moderate[b]

© 2024 American Medical Association. All rights reserved.

Dupilumab is probably associated with a small important reduction in POEM scores vs Tralokinumab.					
Dupilumab	Upadacitinib 15 mg daily	0	--	-0.3 (-4.4, 3.9); high	-0.3 (-4.4, 3.9); low[c]
Dupilumab may be associated with no important difference in reduction in POEM scores vs Upadacitinib 15 mg daily.					
Dupilumab	Upadacitinib 30 mg daily	0	--	3.3 (-0.9, 7.5); high	3.3 (-0.9, 7.5); low[c]
Upadacitinib 30 mg daily may be associated with a small important reduction in POEM scores vs Dupilumab.					
Lebrikizumab	Placebo	4	-6.1 (-7.6, -4.7); high	--	-6.2 (-7.4, -5.1); high
Lebrikizumab is associated with a large important reduction in POEM scores vs placebo.					
Lebrikizumab	Tralokinumab	0	--	-2 (-3.5, -0.6); high	-2 (-3.5, -0.6); moderate[b]
Lebrikizumab is probably associated with a small important reduction in POEM scores vs Tralokinumab.					
Lebrikizumab	Upadacitinib 15 mg daily	0	--	0.7 (-3.5, 5.1); high	0.7 (-3.5, 5.1); low[c]
Lebrikizumab may be associated with no important difference in reduction in POEM scores vs Upadacitinib 15 mg daily.					
Lebrikizumab	Upadacitinib 30 mg daily	0	--	4.4 (0.2, 8.8); high	4.4 (0.2, 8.8); low[c]
Upadacitinib 30 mg daily may be associated with a large important reduction in POEM scores vs Lebrikizumab.					
Tralokinumab	Placebo	3	-4.2 (-5.0, -3.5); high	--	-4.2 (-5, -3.4); high
Tralokinumab is associated with a large important reduction in POEM scores vs placebo.					
Tralokinumab	Upadacitinib 15 mg daily	0	--	2.8 (-1.3, 7.1); high	2.8 (-1.3, 7.1); low[c]
Upadacitinib 15 mg daily may be associated with a small important reduction in POEM scores vs Tralokinumab.					
Tralokinumab	Upadacitinib 30 mg daily	0	--	6.4 (2.2, 10.7); high	6.4 (2.2, 10.7); moderate[b]
Upadacitinib 30 mg daily is probably associated with a large important reduction in POEM scores vs Tralokinumab.					
Upadacitinib 15 mg daily	Placebo	1	-7.0 (-11.0, -3.0); high	--	-7 (-11.2, -2.9); moderate[b]
Upadacitinib 15 mg daily is probably associated with a large important reduction in POEM scores vs placebo.					
Upadacitinib 15 mg daily	Upadacitinib 30 mg daily	1	3.7 (-0.2, 7.6); high	--	3.7 (-0.3, 7.7); low[c]
Upadacitinib 30 mg daily may be associated with a large important reduction in POEM scores vs Upadacitinib 15 mg daily.					
Upadacitinib 30 mg daily	Placebo	1	-10.7 (-14.7, -6.7); high	--	-10.7 (-14.8, -6.5); high
Upadacitinib 30 mg daily is associated with a large important reduction in POEM scores vs placebo.					

Dose of dupilumab and tralokinumab is 600 mg then 300 mg every 2 weeks.
Dose of lebrikizumab is 500 mg at weeks 0 and 2 then 250 mg every 2 weeks.

CI: Confidence interval. CrI: Credible interval.

The minimal important difference for POEM is 3.4.[2]

*Direct estimates, when available, are derived from random-effects frequentist pairwise meta-analysis.
^Indirect effects are taken from node splitting analyses when available; otherwise, the network effect is used.

© 2024 American Medical Association. All rights reserved.

a. Rated down for inconsistency
b. Rated down for imprecision
c. Rated down two levels for major imprecision

© 2024 American Medical Association. All rights reserved.

eFigure 3. Network plot of studies included in the network meta-analysis of adults* treated between 8 and 16 weeks for change in Dermatology Life Quality Index (DLQI)

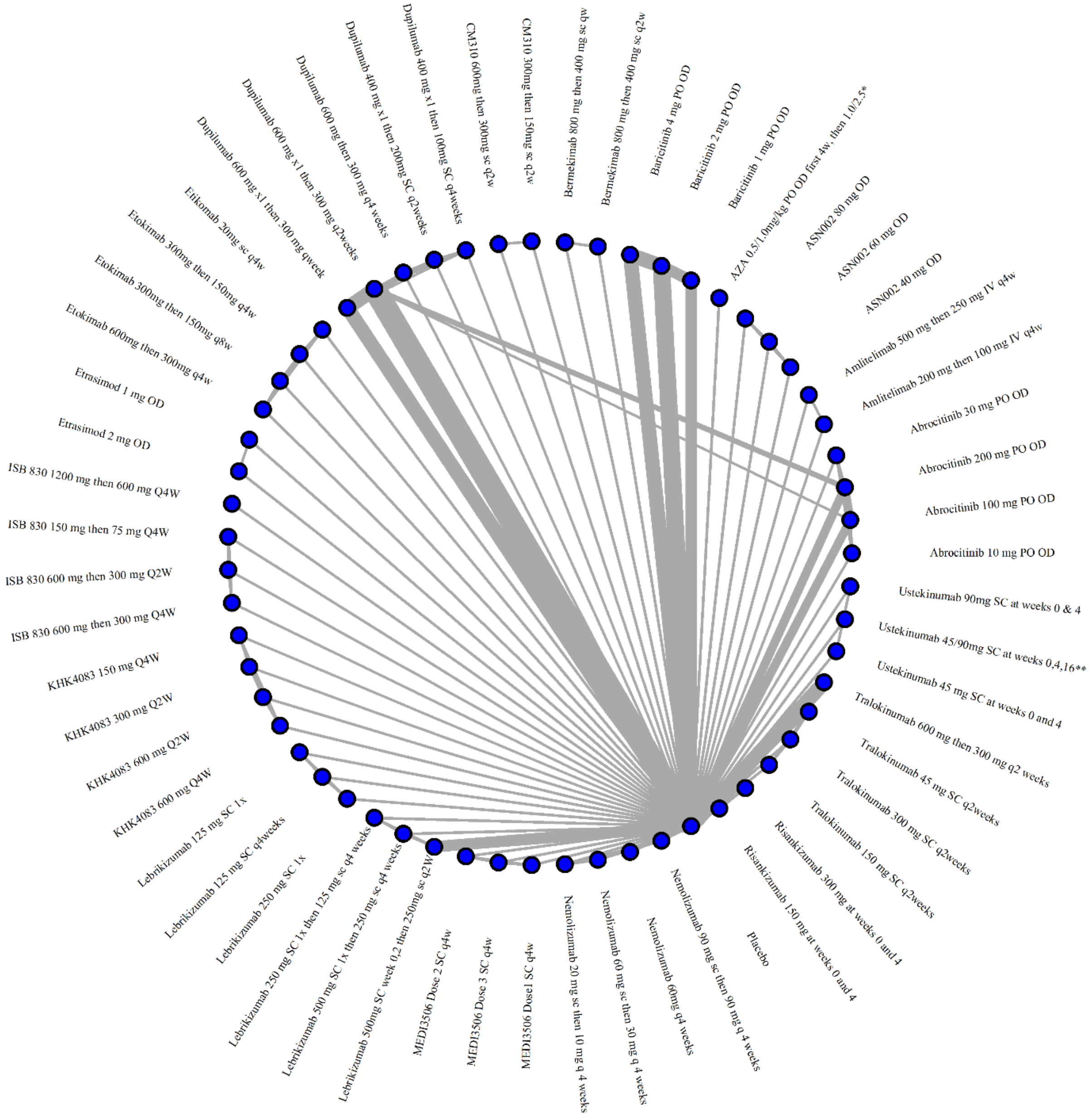

* Azathioprine 0.5 mg/kg or 1.0 mg/kg PO OD first 4 weeks, then 1.0 mg/kg or 2.5 mg/kg PO OD
** Weight-based dosing

The width of each line connecting two treatments (nodes) is proportional to the number of head-to-head trials for that comparison.

OD: once daily; q1w: once weekly; q2w: every 2 weeks; q4w: every 4 weeks.

*Some studies included in the analyses of trials of adults include a minority proportion of adolescent (12-17 years old) participants

© 2024 American Medical Association. All rights reserved.

eTable 5. Relative effect estimates for change in DLQI up to 16 weeks of treatment in adults for placebo and medications used in clinical practice. Results for other pairwise comparisons in the network can be requested from the authors. Results are presented as change in DLQI (95% CrI). A positive effect estimate in a given cell favors the row-defining treatment. A negative effect estimate in a given cell favors the column-defining treatment. The bottom row contains the Surface Under the Cumulative Ranking (SUCRA) value for the column-defining treatment.

	Abro 100	Abro 200	Bari 2	Bari 4	Dupi	Lebri	Placebo	Tralo
Abro 100		-2.1 (-2.9, -1.2)	1.2 (0, 2.4)	-0.1 (-1.3, 1.2)	-1.4 (-2.3, -0.5)	-1.2 (-3.2, 0.8)	3.4 (2.6, 4.3)	1.1 (0, 2.2)
Abro 200	2.1 (1.2, 2.9)		3.3 (2.1, 4.4)	2 (0.8, 3.2)	0.7 (-0.1, 1.4)	0.9 (-1, 2.9)	5.6 (4.8, 6.3)	3.2 (2.1, 4.2)
Bari 2	-1.2 (-2.4, 0)	-3.3 (-4.4, -2.1)		-1.3 (-2.2, -0.3)	-2.6 (-3.6, -1.6)	-2.4 (-4.3, -0.4)	2.3 (1.4, 3.1)	-0.1 (-1.2, 1)
Bari 4	0.1 (-1.2, 1.3)	-2 (-3.2, -0.8)	1.3 (0.3, 2.2)		-1.3 (-2.4, -0.3)	-1.1 (-3.1, 0.9)	3.5 (2.6, 4.4)	1.2 (0, 2.3)
Dupi	1.4 (0.5, 2.3)	-0.7 (-1.4, 0.1)	2.6 (1.6, 3.6)	1.3 (0.3, 2.4)		0.2 (-1.6, 2.1)	4.9 (4.3, 5.4)	2.5 (1.6, 3.4)
Lebri	1.2 (-0.8, 3.2)	-0.9 (-2.9, 1)	2.4 (0.4, 4.3)	1.1 (-0.9, 3.1)	-0.2 (-2.1, 1.6)		4.7 (2.8, 6.4)	2.3 (0.4, 4.2)
Placebo	-3.4 (-4.3, -2.6)	-5.6 (-6.3, -4.8)	-2.3 (-3.1, -1.4)	-3.5 (-4.4, -2.6)	-4.9 (-5.4, -4.3)	-4.7 (-6.4, -2.8)		-2.4 (-3.1, -1.6)
Tralo	-1.1 (-2.2, 0)	-3.2 (-4.2, -2.1)	0.1 (-1, 1.2)	-1.2 (-2.3, 0)	-2.5 (-3.4, -1.6)	-2.3 (-4.2, -0.4)	2.4 (1.6, 3.1)	
SUCRA	0.73	0.94	0.57	0.74	0.87	0.85	0.22	0.58

Abro 100/200: abrocitinib 100 mg/200 mg daily; bari 2/4: baricitinib 2mg/4 mg daily; dupi: dupilumab 600 mg then 300 mg every 2 weeks; lebri: lebrikizumab 500 mg at week 0 and 2 then 250 mg every 2 weeks; tralo: tralokinumab 600 mg then 300 mg every 2 weeks; upa 15/30: upadacitinib 15mg/30 mg daily.

© 2024 American Medical Association. All rights reserved.

eTable 6. GRADE certainty ratings for change in DLQI up to 16 weeks of treatment in adults for placebo and medications used in clinical practice. Results are presented as change in EASI (95% CrI). Negative effect estimates favor the treatment listed in the intervention column; positive effect estimates favor the comparator.

Intervention	Comparator	Head-to-head trials, n	Direct estimate (95% CI); Certainty of Evidence*	Indirect estimate (95% CrI); Certainty of Evidence^	NMA Estimate (95% CrI); Certainty of Evidence
Abrocitinib 100 mg daily	Abrocitinib 200 mg daily	4	2.0 (1.2, 2.9); moderate[a]	--	2.1 (1.2, 2.9); low[a,b]
Abrocitinib 200 mg daily may be associated with a small important reduction in DLQI scores vs Abrocitinib 100 mg daily.					
Abrocitinib 100 mg daily	Baricitinib 2 mg daily	0	--	-1.2 (-2.4, 0); high	-1.2 (-2.4, 0); moderate[b]
Abrocitinib 100 mg daily is probably associated with no important difference in reduction in DLQI scores vs Baricitinib 2 mg daily.					
Abrocitinib 100 mg daily	Baricitinib 4 mg daily	0	--	0.1 (-1.2, 1.3); high	0.1 (-1.2, 1.3); high
Abrocitinib 100 mg daily is associated with no important difference in reduction in DLQI scores vs Baricitinib 4 mg daily.					
Abrocitinib 100 mg daily	Dupilumab	1	1.8 (0.9, 2.7); high	0.9 (-0.3, 2.2); high	1.4 (0.5, 2.3); moderate[b]
Abrocitinib 100 mg daily is probably associated with no important difference in reduction in DLQI scores vs Dupilumab.					
Abrocitinib 100 mg daily	Lebrikizumab	0	--	1.2 (-0.8, 3.2); high	1.2 (-0.8, 3.2); moderate[b]
Abrocitinib 100 mg daily is probably associated with no important difference in reduction in DLQI scores vs Lebrikizumab.					
Abrocitinib 100 mg daily	Placebo	4	-3.4 (-4.4, -2.4); high	--	-3.4 (-4.3, -2.6); moderate[b]
Abrocitinib 100 mg daily is probably associated with a large important reduction in DLQI scores vs Placebo.					
Abrocitinib 100 mg daily	Tralokinumab	0	--	-1.1 (-2.2, 0); high	-1.1 (-2.2, 0); moderate[b]
Abrocitinib 100 mg daily is probably associated with no important difference in reduction in DLQI scores vs Tralokinumab.					
Abrocitinib 200 mg daily	Baricitinib 2 mg daily	0	--	-3.3 (-4.4, -2.1); high	-3.3 (-4.4, -2.1); moderate[b]
Abrocitinib 200 mg daily is probably associated with a large important reduction in DLQI scores vs Baricitinib 2 mg daily.					
Abrocitinib 200 mg daily	Baricitinib 4 mg daily	0	--	-2 (-3.2, -0.8); high	-2 (-3.2, -0.8); moderate[b]
Abrocitinib 200 mg daily is probably associated with a small important reduction in DLQI scores vs Baricitinib 4 mg daily.					
Abrocitinib 200 mg daily	Dupilumab	1	-0.8 (-1.3, 0.3); high	-0.1 (-1.6, 1.3); high	-0.7 (-1.4, 0.1); high
Abrocitinib 200 mg daily is associated with no important difference in reduction in DLQI scores vs Dupilumab.					
Abrocitinib 200 mg daily	Lebrikizumab	0	--	-0.9 (-2.9, 1); high	-0.9 (-2.9, 1); moderate[b]
Abrocitinib 200 mg daily is probably associated with no important difference in reduction in DLQI scores vs Lebrikizumab.					
Abrocitinib 200 mg daily	Placebo	4	-5.5 (-6.3, -4.6); high	-5.7 (-7.1, -4.3); high	-5.6 (-6.3, -4.8); high
Abrocitinib 200 mg daily is associated with a large important reduction in DLQI scores vs Placebo.					
Abrocitinib 200 mg daily	Tralokinumab	0	--	-3.2 (-4.2, -2.1); high	-3.2 (-4.2, -2.1); moderate[b]
Abrocitinib 200 mg daily is probably associated with a small important reduction in DLQI scores vs Tralokinumab.					
Baricitinib 2 mg daily	Baricitinib 4 mg daily	5	1.3 (0.5, 2.2); high	--	1.3 (0.3, 2.2); moderate[b]
Baricitinib 2 mg daily is probably associated with no important difference in reduction in DLQI scores vs Baricitinib 4 mg daily.					

© 2024 American Medical Association. All rights reserved.

Baricitinib 2 mg daily	Dupilumab	0	--	2.6 (1.6, 3.6); high	2.6 (1.6, 3.6); moderate[b]
Dupilumab is probably associated with a small important reduction in DLQI scores vs Baricitinib 2 mg daily.					
Baricitinib 2 mg daily	Lebrikizumab	0	--	2.4 (0.4, 4.3); high	2.4 (0.4, 4.3); moderate[b]
Lebrikizumab is probably associated with a small important reduction in DLQI scores vs Baricitinib 2 mg daily.					
Baricitinib 2 mg daily	Placebo	6	-2.3 (-3.2, -1.3); high	--	-2.3 (-3.1, -1.4); moderate[b]
Baricitinib 2 mg daily is probably associated with a small important reduction in DLQI scores vs placebo.					
Baricitinib 2 mg daily	Tralokinumab	0	--	0.1 (-1, 1.2); high	0.1 (-1, 1.2); high
Baricitinib 2 mg daily is associated with no important difference in reduction in DLQI scores vs Tralokinumab.					
Baricitinib 4 mg daily	Dupilumab	0	--	1.3 (0.3, 2.4); high	1.3 (0.3, 2.4); moderate[b]
Baricitinib 4 mg daily is probably associated with no important difference in reduction in DLQI scores vs Dupilumab.					
Baricitinib 4 mg daily	Lebrikizumab	0	--	1.1 (-0.9, 3.1); high	1.1 (-0.9, 3.1); moderate[b]
Baricitinib 4 mg daily is probably associated with no important difference in reduction in DLQI scores vs Lebrikizumab.					
Baricitinib 4 mg daily	Placebo	5	-3.5 (-4.3, -2.7); high	--	-3.5 (-4.4, -2.6); moderate[b]
Baricitinib 4 mg daily is probably associated with a large important reduction in DLQI scores vs placebo.					
Baricitinib 4 mg daily	Tralokinumab	0	--	-1.2 (-2.3, 0); high	-1.2 (-2.3, 0); moderate[b]
Baricitinib 4 mg daily is probably associated with no important difference in reduction in DLQI scores vs Tralokinumab.					
Dupilumab	Lebrikizumab	0	--	-0.2 (-2.1, 1.6); high	-0.2 (-2.1, 1.6); moderate[b]
Dupilumab is probably associated with no important difference in reduction in DLQI scores vs Lebrikizumab.					
Dupilumab	Placebo	8	-4.8 (-5.3, -4.3); high	-4.9 (-6.5, -3.1); high	-4.9 (-5.4, -4.3); high
Dupilumab is associated with a large important reduction in DLQI scores vs placebo.					
Dupilumab	Tralokinumab	0	--	-2.5 (-3.4, -1.6); high	-2.5 (-3.4, -1.6); moderate[b]
Dupilumab may be associated with a small important reduction in DLQI scores vs Tralokinumab.					
Lebrikizumab	Placebo	4	-6.6 (-6.3, -2.9); high	--	-4.7 (-6.4, -2.8); moderate[b]
Lebrikizumab is probably associated with a large important reduction in DLQI scores vs Placebo.					
Lebrikizumab	Tralokinumab	0	--	-2.3 (-4.2, -0.4); high	-2.3 (-4.2, -0.4); moderate[b]
Lebrikizumab is probably associated with a small important reduction in DLQI scores vs Tralokinumab.					
Tralokinumab	Placebo	5	-2.4 (-3.3, -1.4); high	--	-2.4 (-3.1, -1.6); moderate[b]
Tralokinumab is probably associated with a small important reduction in DLQI scores vs Placebo.					

Dose of dupilumab and tralokinumab is 600 mg then 300 mg every 2 weeks.
Dose of lebrikizumab is 500 mg at weeks 0 and 2 then 250 mg every 2 weeks.

CI: Confidence interval. CrI: Credible interval.

The minimal important difference for DLQI is 3.3.[4]

© 2024 American Medical Association. All rights reserved.

*Direct estimates, when available, are derived from random-effects frequentist pairwise meta-analysis.
^Indirect effects are taken from node splitting analyses when available; otherwise, the network effect is used.

a. Rated down for inconsistency
b. Rated down for imprecision

© 2024 American Medical Association. All rights reserved.

eFigure 4. Network plot of studies included in the network meta-analysis of adults* treated between 8 and 16 weeks for change in Peak Pruritus Numeric Rating Scale (PP-NRS)

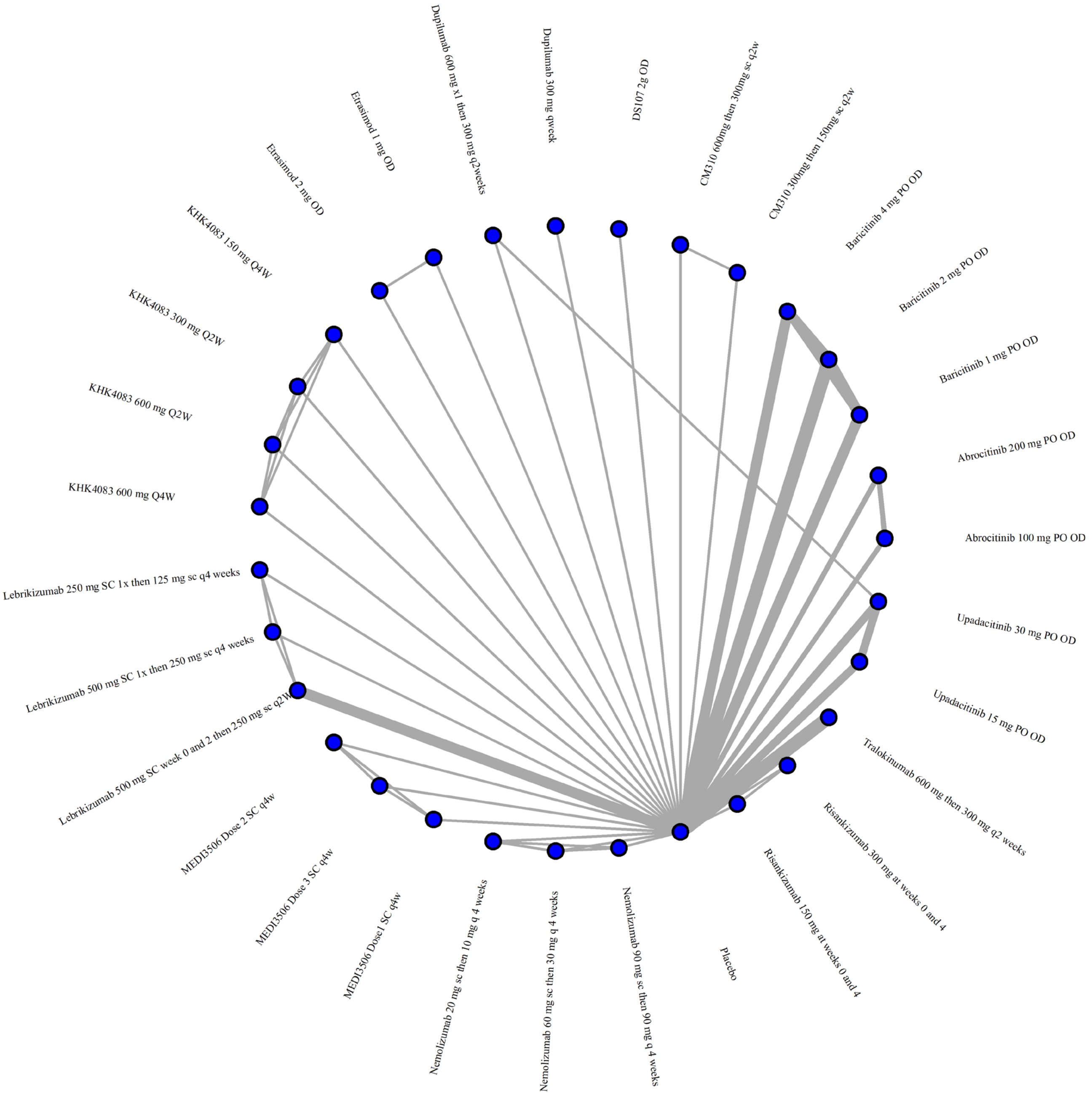

The width of each line connecting two treatments (nodes) is proportional to the number of head-to-head trials for that comparison.

OD: once daily; q1w: once weekly; q2w: every 2 weeks; q4w: every 4 weeks.

*Some studies included in the analyses of trials of adults include a minority proportion of adolescent (12-17 years old) participants

© 2024 American Medical Association. All rights reserved.

eTable 7. Relative effect estimates for change in peak pruritus numeric rating scales up to 16 weeks of treatment in adults for placebo and medications used in clinical practice. Results for other pairwise comparisons in the network can be requested from the authors. Results are presented as change in the numeric rating scales (95% CrI). A positive effect estimate in a given cell favors the row-defining treatment. A negative effect estimate in a given cell favors the column-defining treatment. The bottom row contains the Surface Under the Cumulative Ranking (SUCRA) value for the column-defining treatment.

	Abro 100	Abro 200	Bari 2	Bari 4	Dupi	Lebri	Placebo	Tralo	Upa 15	Upa 30
Abro 100		-0.8 (-1.3, -0.4)	0.4 (-0.2, 1.1)	0 (-0.7, 0.6)	-0.5 (-1, 0.1)	-0.5 (-1.2, 0.2)	1.6 (1.1, 2.1)	0.6 (0, 1.3)	-0.8 (-1.5, -0.1)	-1.7 (-2.4, -1.1)
Abro 200	0.8 (0.4, 1.3)		1.2 (0.6, 1.9)	0.8 (0.1, 1.4)	0.4 (-0.3, 1)	0.3 (-0.4, 1)	2.4 (1.8, 3)	1.5 (0.8, 2.1)	0 (-0.6, 0.7)	-0.9 (-1.6, -0.3)
Bari 2	-0.4 (-1.1, 0.2)	-1.2 (-1.9, -0.6)		-0.5 (-0.8, -0.1)	-0.9 (-1.3, -0.4)	-0.9 (-1.5, -0.4)	1.2 (0.9, 1.6)	0.2 (-0.2, 0.7)	-1.2 (-1.7, -0.7)	-2.2 (-2.6, -1.7)
Bari 4	0 (-0.6, 0.7)	-0.8 (-1.4, -0.1)	0.5 (0.1, 0.8)		-0.4 (-0.8, 0)	-0.5 (-1.1, 0.1)	1.6 (1.3, 2)	0.7 (0.2, 1.2)	-0.7 (-1.3, -0.2)	-1.7 (-2.2, -1.2)
Dupi	0.5 (-0.1, 1)	-0.4 (-1, 0.3)	0.9 (0.4, 1.3)	0.4 (0, 0.8)		-0.1 (-0.6, 0.4)	2.1 (1.8, 2.3)	1.1 (0.8, 1.4)	-0.3 (-0.7, 0)	-1.3 (-1.5, -1.1)
Lebri	0.5 (-0.2, 1.2)	-0.3 (-1, 0.4)	0.9 (0.4, 1.5)	0.5 (-0.1, 1.1)	0.1 (-0.4, 0.6)		2.1 (1.7, 2.6)	1.2 (0.6, 1.7)	-0.3 (-0.8, 0.3)	-1.2 (-1.7, -0.7)
Placebo	-1.6 (-2.1, -1.1)	-2.4 (-3, -1.8)	-1.2 (-1.6, -0.9)	-1.6 (-2, -1.3)	-2.1 (-2.3, -1.8)	-2.1 (-2.6, -1.7)		-1 (-1.2, -0.7)	-2.4 (-2.8, -2)	-3.3 (-3.6, -3.1)
Tralo	-0.6 (-1.3, 0)	-1.5 (-2.1, -0.8)	-0.2 (-0.7, 0.2)	-0.7 (-1.2, -0.2)	-1.1 (-1.4, -0.8)	-1.2 (-1.7, -0.6)	1 (0.7, 1.2)		-1.4 (-1.9, -1)	-2.4 (-2.8, -2)
Upa 15	0.8 (0.1, 1.5)	0 (-0.7, 0.6)	1.2 (0.7, 1.7)	0.7 (0.2, 1.3)	0.3 (0, 0.7)	0.3 (-0.3, 0.8)	2.4 (2, 2.8)	1.4 (1, 1.9)		-1 (-1.3, -0.6)
Upa 30	1.7 (1.1, 2.4)	0.9 (0.3, 1.6)	2.2 (1.7, 2.6)	1.7 (1.2, 2.2)	1.3 (1.1, 1.5)	1.2 (0.7, 1.7)	3.3 (3.1, 3.6)	2.4 (2, 2.8)	1 (0.6, 1.3)	
SUCRA	0.56	0.84	0.44	0.58	0.72	0.75	0.09	0.35	0.84	0.99

Abro 100/200: abrocitinib 100 mg/200 mg daily; bari 2/4: baricitinib 2mg/4 mg daily; dupi: dupilumab 600 mg then 300 mg every 2 weeks; lebri: lebrikizumab 500 mg at week 0 and 2 then 250 mg every 2 weeks; tralo: tralokinumab 600 mg then 300 mg every 2 weeks; upa 15/30: upadacitinib 15mg/30 mg daily.

© 2024 American Medical Association. All rights reserved.

eTable 8. GRADE certainty ratings for change in peak pruritus numeric rating scales (PP-NRS) up to 16 weeks of treatment in adults for placebo and medications used in clinical practice or likely to be approved soon. Results are presented as change in the numeric rating scales (95% CrI). Negative effect estimates favor the treatment listed in the intervention column; positive effect estimates favor the comparator.

Intervention	Comparator	Head-to-head trials, n	Direct estimate (95% CI); Certainty of Evidence*	Indirect estimate (95% CrI); Certainty of Evidence^	NMA Estimate (95% CrI); Certainty of Evidence
Abrocitinib 100 mg daily	Abrocitinib 200 mg daily	3	0.8 (0.4, 1.3); high	--	0.8 (0.4, 1.3); moderate[b]
Abrocitinib 100 mg daily is probably associated with no important difference in reduction in PP-NRS scores vs Abrocitinib 200 mg daily.					
Abrocitinib 100 mg daily	Baricitinib 2 mg daily	0	--	-0.4 (-1.1, 0.2); high	-0.4 (-1.1, 0.2); high
Abrocitinib 100 mg daily is associated with no important difference in reduction in PP-NRS scores vs Baricitinib 2 mg daily.					
Abrocitinib 100 mg daily	Baricitinib 4 mg daily	0	--	0 (-0.6, 0.7); high	0 (-0.6, 0.7); high
Abrocitinib 100 mg daily is associated with no important difference in reduction in PP-NRS scores vs Baricitinib 4 mg daily.					
Abrocitinib 100 mg daily	Dupilumab	0	--	0.5 (-0.1, 1); high	0.5 (-0.1, 1); high
Abrocitinib 100 mg daily is associated with no important difference in reduction in PP-NRS scores vs Dupilumab.					
Abrocitinib 100 mg daily	Lebrikizumab	0	--	0.5 (-0.2, 1.2); moderate[a]	0.5 (-0.2, 1.2); moderate[a]
Abrocitinib 100 mg daily is probably associated with no important difference in reduction in PP-NRS scores vs Lebrikizumab.					
Abrocitinib 100 mg daily	Placebo	3	-1.6 (-2.1, -1); high	--	-1.6 (-2.1, -1.1); moderate[b]
Abrocitinib 100 mg daily is probably associated with a small important reduction in PP-NRS scores vs Placebo.					
Abrocitinib 100 mg daily	Tralokinumab	0	--	-0.6 (-1.3, 0); moderate[a]	-0.6 (-1.3, 0); low[a,b]
Abrocitinib 100 mg daily may be associated with no important difference in reduction in PP-NRS scores vs Tralokinumab.					
Abrocitinib 100 mg daily	Upadacitinib 15 mg daily	0	--	0.8 (0.1, 1.5); high	0.8 (0.1, 1.5); moderate[b]
Abrocitinib 100 mg daily is probably associated with no important difference in reduction in PP-NRS scores vs Upadacitinib 15 mg daily.					
Abrocitinib 100 mg daily	Upadacitinib 30 mg daily	0	--	1.7 (1.1, 2.4); high	1.7 (1.1, 2.4); moderate[b]
Upadacitinib 30 mg daily is probably associated with a small important reduction in PP-NRS scores vs Abrocitinib 100 mg daily.					
Abrocitinib 200 mg daily	Baricitinib 2 mg daily	0	--	-1.2 (-1.9, -0.6); high	-1.2 (-1.9, -0.6); moderate[b]
Abrocitinib 200 mg daily is probably associated with no important difference in reduction in PP-NRS scores vs Baricitinib 2 mg daily.					
Abrocitinib 200 mg daily	Baricitinib 4 mg daily	0	--	-0.7 (-1.4, 0); high	-0.8 (-1.4, -0.1); moderate[b]
Abrocitinib 200 mg daily is probably associated with no important difference in reduction in PP-NRS scores vs Baricitinib 4 mg daily.					
Abrocitinib 200 mg daily	Dupilumab	0	--	-0.4 (-1, 0.3); high	-0.4 (-1, 0.3); high
Abrocitinib 200 mg daily is associated with no important difference in reduction in PP-NRS scores vs Dupilumab.					
Abrocitinib 200 mg daily	Lebrikizumab	0	--	-0.3 (-1, 0.4); moderate[a]	-0.3 (-1, 0.4); moderate[a]
Abrocitinib 200 mg daily is probably associated with no important difference in reduction in PP-NRS scores vs Lebrikizumab.					
Abrocitinib 200 mg daily	Placebo	3	-2.4 (-3.0, -1.9); high	--	-2.4 (-3, -1.8); moderate[b]
Abrocitinib 200 mg daily is probably associated with a small important reduction in PP-NRS scores vs Placebo.					

© 2024 American Medical Association. All rights reserved.

Abrocitinib 200 mg daily	Tralokinumab	0	--	-1.5 (-2.1, -0.8); moderate[a]	-1.5 (-2.1, -0.8); low[a,b]
Abrocitinib 200 mg daily may be associated with a small important reduction in PP-NRS scores vs Tralokinumab.					
Abrocitinib 200 mg daily	Upadacitinib 15 mg daily	0	--	0 (-0.7, 0.6); high	0 (-0.7, 0.6); high
Abrocitinib 200 mg daily is associated with no important difference in reduction in PP-NRS scores vs Upadacitinib 15 mg daily.					
Abrocitinib 200 mg daily	Upadacitinib 30 mg daily	0	--	1 (0.3, 1.7); high	0.9 (0.3, 1.6); moderate[b]
Abrocitinib 200 mg daily is probably associated with no important difference in reduction in PP-NRS scores vs Upadacitinib 30 mg daily.					
Baricitinib 2 mg daily	Baricitinib 4 mg daily	4	0.5 (0.1, 0.8); high	--	0.5 (0.1, 0.8); high
Baricitinib 2 mg daily is associated with no important difference in reduction in PP-NRS scores vs Baricitinib 4 mg daily.					
Baricitinib 2 mg daily	Dupilumab	0	--	0.9 (0.4, 1.3); high	0.9 (0.4, 1.3); moderate[b]
Baricitinib 2 mg daily is probably associated with no important difference in reduction in PP-NRS scores vs Dupilumab.					
Baricitinib 2 mg daily	Lebrikizumab	0	--	0.9 (0.4, 1.5); moderate[a]	0.9 (0.4, 1.5); low[a,b]
Baricitinib 2 mg daily may be associated with no important difference in reduction in PP-NRS scores vs Lebrikizumab.					
Baricitinib 2 mg daily	Placebo	5	-1.2 (-1.5, -0.9); high	--	-1.2 (-1.6, -0.9); moderate[b]
Baricitinib 2 mg daily is probably associated with no important difference in reduction in PP-NRS scores vs Placebo.					
Baricitinib 2 mg daily	Tralokinumab	0	--	-0.2 (-0.7, 0.2); moderate[a]	-0.2 (-0.7, 0.2); moderate[a]
Baricitinib 2 mg daily is probably associated with no important difference in reduction in PP-NRS scores vs Tralokinumab.					
Baricitinib 2 mg daily	Upadacitinib 15 mg daily	0	--	1.2 (0.7, 1.7); high	1.2 (0.7, 1.7); moderate[b]
Baricitinib 2 mg daily is probably associated with no important difference in reduction in PP-NRS scores vs Upadacitinib 15 mg daily.					
Baricitinib 2 mg daily	Upadacitinib 30 mg daily	0	--	2.2 (1.7, 2.6); high	2.2 (1.7, 2.6); moderate[b]
Upadacitinib 30 mg daily is probably associated with a small important reduction in PP-NRS scores vs Baricitinib 2 mg daily.					
Baricitinib 4 mg daily	Dupilumab	0	--	0.4 (0, 0.8); high	0.4 (0, 0.8); high
Baricitinib 4 mg daily is associated with no important difference in reduction in PP-NRS scores vs Dupilumab.					
Baricitinib 4 mg daily	Lebrikizumab	0	--	0.5 (-0.1, 1.1); moderate[a]	0.5 (-0.1, 1.1); moderate[a]
Baricitinib 4 mg daily is probably associated with no important difference in reduction in PP-NRS scores vs Lebrikizumab.					
Baricitinib 4 mg daily	Placebo	4	-1.6 (-2.0, -1.3); high	--	-1.6 (-2, -1.3); high
Baricitinib 4 mg daily is associated with a small important reduction in PP-NRS scores vs placebo.					
Baricitinib 4 mg daily	Tralokinumab	0	--	-0.7 (-1.2, -0.2); moderate[a]	-0.7 (-1.2, -0.2); moderate[a]
Baricitinib 4 mg daily is probably associated with no important difference in reduction in PP-NRS scores vs Tralokinumab.					
Baricitinib 4 mg daily	Upadacitinib 15 mg daily	0	--	0.7 (0.2, 1.3); high	0.7 (0.2, 1.3); moderate[b]
Baricitinib 4 mg daily is probably associated with no important difference in reduction in PP-NRS scores vs Upadacitinib 15 mg daily.					
Baricitinib 4 mg daily	Upadacitinib 30 mg daily	0	--	1.7 (1.2, 2.2); high	1.7 (1.2, 2.2); moderate[b]
Upadacitinib 30 mg daily is probably associated with a small important reduction in PP-NRS scores vs Baricitinib 4 mg daily.					
Dupilumab	Lebrikizumab	0	--	0.1 (-0.4, 0.6); moderate[a]	0.1 (-0.4, 0.6); moderate[a]
Dupilumab is probably associated with no important difference in reduction in PP-NRS scores vs Lebrikizumab.					
Dupilumab	Placebo	7	-2.0 (-2.3, -1.8); high	-2.2 (-2.6, -1.7); high	-2.1 (-2.3, -1.8); high

© 2024 American Medical Association. All rights reserved.

Dupilumab is associated with a small important reduction in PP-NRS scores vs placebo.					
Dupilumab	Tralokinumab	0	--	-1.1 (-1.4, -0.8); moderate[a]	-1.1 (-1.4, -0.8); low[a,b]
Dupilumab may be associated with no important difference in reduction in PP-NRS scores vs Tralokinumab.					
Dupilumab	Upadacitinib 15 mg daily	0	--	0.3 (0, 0.7); high	0.3 (0, 0.7); high
Dupilumab is associated with no important difference in reduction in PP-NRS scores vs Upadacitinib 15 mg daily.					
Dupilumab	Upadacitinib 30 mg daily	1	1.3 (1.3, 1.3); high	1.4 (0.9, 1.9); high	1.3 (1.1, 1.5); moderate[b]
Upadacitinib 30 mg daily is probably associated with a small important reduction in PP-NRS scores vs Dupilumab.					
Lebrikizumab	Placebo	4	-2.4 (-3.5, -1.2); moderate[a]	--	-2.1 (-2.6, -1.7); low[a,b]
Lebrikizumab may be associated with a small important reduction in PP-NRS scores vs Placebo.					
Lebrikizumab	Tralokinumab	0	--	-1.2 (-1.7, -0.6); moderate[a]	-1.2 (-1.7, -0.6) ; low[a,b]
Lebrikizumab may be associated with no important difference in reduction in PP-NRS scores vs Tralokinumab.					
Lebrikizumab	Upadacitinib 15 mg daily	0	--	0.3 (-0.3, 0.8); moderate[a]	0.3 (-0.3, 0.8); moderate[a]
Lebrikizumab is probably associated with no important difference in reduction in PP-NRS scores vs Upadacitinib 15 mg daily.					
Lebrikizumab	Upadacitinib 30 mg daily	0	--	1.2 (0.7, 1.7); moderate[a]	1.2 (0.7, 1.7); low[a,b]
Lebrikizumab may be associated with no important difference in reduction in PP-NRS scores vs Upadacitinib 30 mg daily.					
Tralokinumab	Placebo	5	-0.9 (-1.3, -0.5); moderate[a]	--	-1 (-1.2, -0.7); moderate[a]
Tralokinumab is probably associated with no important difference in reduction in PP-NRS scores vs Placebo.					
Tralokinumab	Upadacitinib 15 mg daily	0	--	1.4 (1, 1.9); moderate[a]	1.4 (1, 1.9); low[a,b]
Upadacitinib 15 mg daily may be associated with a small important reduction in PP-NRS scores vs Tralokinumab.					
Tralokinumab	Upadacitinib 30 mg daily	0	--	2.4 (2, 2.8); moderate[a]	2.4 (2, 2.8); low[a,b]
Upadacitinib 30 mg daily may be associated with a small important reduction in PP-NRS scores vs Tralokinumab.					
Upadacitinib 15 mg daily	Placebo	3	-2.4 (-2.8, -2.1); high	--	-2.4 (-2.8, -2); moderate[b]
Upadacitinib 15 mg daily is probably associated with a small important reduction in PP-NRS scores vs Placebo.					
Upadacitinib 15 mg daily	Upadacitinib 30 mg daily	3	1.0 (0.7, 1.3); high	--	1 (0.6, 1.3); moderate[b]
Upadacitinib 15 mg daily is probably associated with no important difference in reduction in PP-NRS scores vs Upadacitinib 30 mg daily.					
Upadacitinib 30 mg daily	Placebo	3	-3.4 (-3.8, -3); high	-3.3 (-3.6, -3.1); high	-3.3 (-3.6, -3.1); high
Upadacitinib 30 mg daily is associated with a large important reduction in PP-NRS scores vs placebo.					

Dose of dupilumab and tralokinumab is 600 mg then 300 mg every 2 weeks.
Dose of lebrikizumab is 500 mg at weeks 0 and 2 then 250 mg every 2 weeks.

CI: Confidence interval. CrI: Credible interval.

© 2024 American Medical Association. All rights reserved.

The minimal important difference for PP-NRS is 2.6.[3]

*Direct estimates, when available, are derived from random-effects frequentist pairwise meta-analysis.
^Indirect effects are taken from node splitting analyses when available; otherwise, the network effect is used.

a. Rated down for inconsistency
b. Rated down for imprecision

© 2024 American Medical Association. All rights reserved.

eFigure 5. Network plot for success achieving 50% improvement in EASI (EASI-50) up to 16 weeks of treatment in adults*.

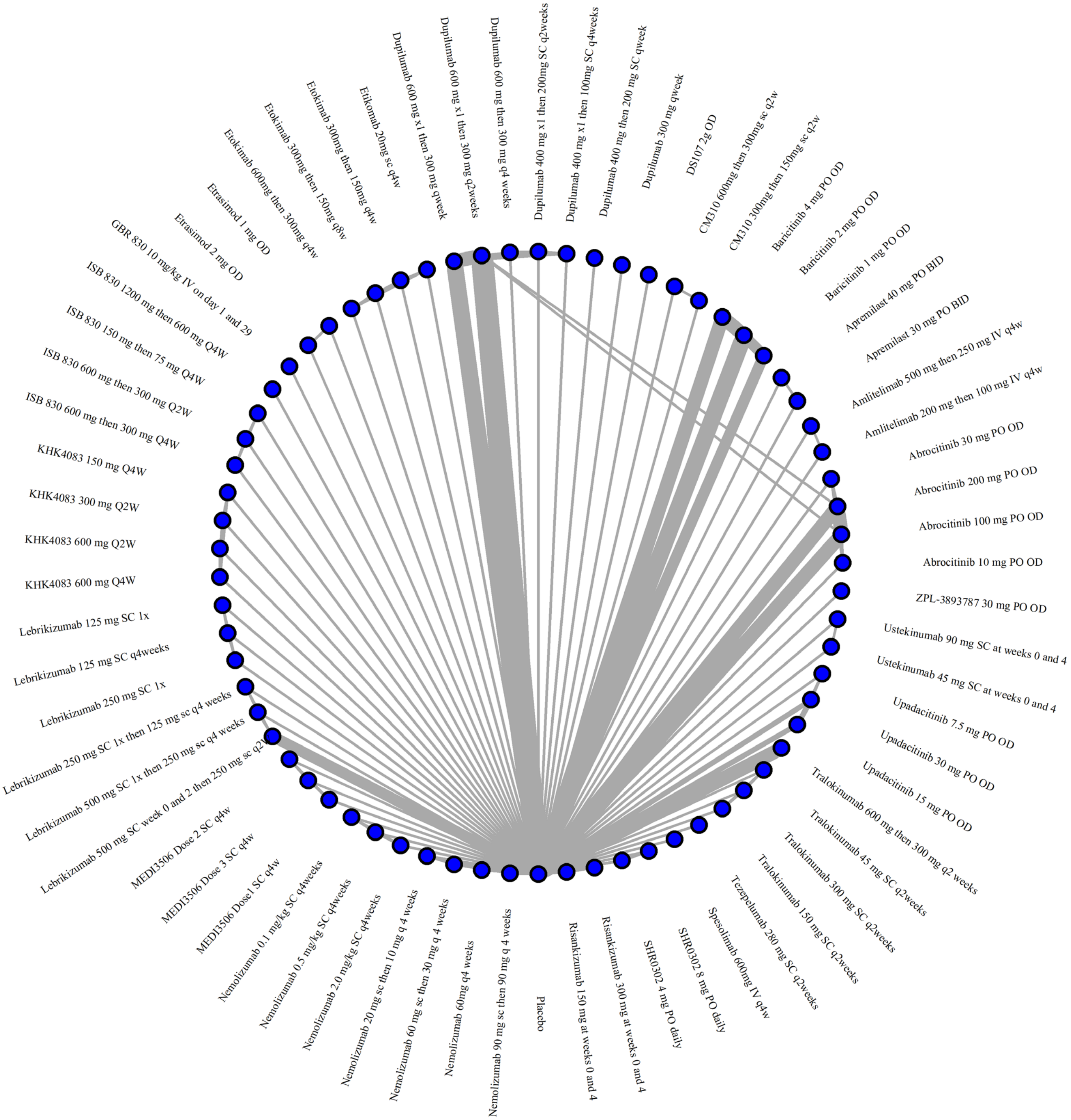

The width of each line connecting two treatments (nodes) is proportional to the number of head-to-head trials for that comparison.
OD: once daily; q1w: once weekly; q2w: every 2 weeks; q4w: every 4 weeks.
*Some studies included in the analyses of trials of adults include a minority proportion of adolescent (12-17 years old) participants.

© 2024 American Medical Association. All rights reserved.

eTable 9. Relative effect estimates for success achieving 50% improvement in EASI (EASI-50) up to 16 weeks of treatment in adults for placebo and medications used in clinical practice. Results for other pairwise comparisons in the network can be requested from the authors. Results are presented as odds ratios (95% credible intervals). Values over 1 indicate greater efficacy for the column-defined treatment. Values less than 1 indicate greater efficacy for the row-defined treatment.

	Abro 100	Abro 200	Bari 2	Bari 4	Dupi	Lebri	Placebo	Tralo	Upa 15	Upa 30
Abro 100		2.2 (1.7, 3)	0.6 (0.4, 0.8)	0.7 (0.5, 1)	1.4 (1, 2)	1 (0.7, 1.5)	0.2 (0.2, 0.3)	0.6 (0.4, 0.9)	2.5 (1.3, 4.9)	3.6 (1.8, 7.6)
Abro 200	0.5 (0.3, 0.6)		0.3 (0.2, 0.4)	0.3 (0.2, 0.5)	0.6 (0.5, 0.9)	0.4 (0.3, 0.7)	0.1 (0.1, 0.1)	0.3 (0.2, 0.4)	1.1 (0.6, 2.2)	1.6 (0.8, 3.4)
Bari 2	1.8 (1.2, 2.6)	4 (2.6, 6)		1.2 (0.9, 1.6)	2.5 (1.8, 3.5)	1.8 (1.2, 2.6)	0.4 (0.3, 0.5)	1.1 (0.8, 1.5)	4.4 (2.3, 8.5)	6.5 (3.3, 13.3)
Bari 4	1.5 (1, 2.2)	3.3 (2.2, 5)	0.8 (0.6, 1.1)		2.1 (1.5, 3)	1.5 (1, 2.2)	0.3 (0.2, 0.4)	0.9 (0.6, 1.3)	3.7 (1.9, 7.1)	5.4 (2.7, 11.1)
Dupi	0.7 (0.5, 1)	1.6 (1.1, 2.2)	0.4 (0.3, 0.5)	0.5 (0.3, 0.7)		0.7 (0.5, 1)	0.2 (0.1, 0.2)	0.4 (0.3, 0.6)	1.7 (0.9, 3.3)	2.6 (1.3, 5.1)
Lebri	1 (0.7, 1.5)	2.2 (1.5, 3.5)	0.6 (0.4, 0.8)	0.7 (0.5, 1)	1.4 (1, 2)		0.2 (0.2, 0.3)	0.6 (0.4, 0.9)	2.5 (1.3, 4.9)	3.7 (1.8, 7.7)
Placebo	4.6 (3.4, 6.1)	10.1 (7.4, 14)	2.6 (2, 3.3)	3.1 (2.3, 4)	6.5 (5.3, 7.9)	4.5 (3.4, 6)		2.7 (2.2, 3.5)	11.3 (6.3, 20.8)	16.6 (8.9, 32.5)
Tralo	1.7 (1.1, 2.4)	3.7 (2.5, 5.5)	0.9 (0.7, 1.3)	1.1 (0.8, 1.6)	2.4 (1.7, 3.3)	1.6 (1.1, 2.4)	0.4 (0.3, 0.5)		4.1 (2.2, 7.9)	6.1 (3.1, 12.5)
Upa 15	0.4 (0.2, 0.8)	0.9 (0.5, 1.7)	0.2 (0.1, 0.4)	0.3 (0.1, 0.5)	0.6 (0.3, 1.1)	0.4 (0.2, 0.8)	0.1 (0, 0.2)	0.2 (0.1, 0.5)		1.5 (0.8, 2.9)
Upa 30	0.3 (0.1, 0.6)	0.6 (0.3, 1.2)	0.2 (0.1, 0.3)	0.2 (0.1, 0.4)	0.4 (0.2, 0.8)	0.3 (0.1, 0.5)	0.1 (0, 0.1)	0.2 (0.1, 0.3)	0.7 (0.3, 1.3)	
SUCRA	0.79	0.95	0.58	0.66	0.89	0.79	0.15	0.61	0.95	0.98

Abro 100/200: abrocitinib 100 mg/200 mg daily; bari 2/4: baricitinib 2mg/4 mg daily; dupi: dupilumab 600 mg then 300 mg every 2 weeks; lebri: lebrikizumab 500 mg at week 0 and 2 then 250 mg every 2 weeks; tralo: tralokinumab 600 mg then 300 mg every 2 weeks; upa 15/30: upadacitinib 15mg/30 mg daily.

© 2024 American Medical Association. All rights reserved.

eFigure 6. Network plot for success achieving 75% improvement in EASI (EASI-75) up to 16 weeks of treatment in adults*.

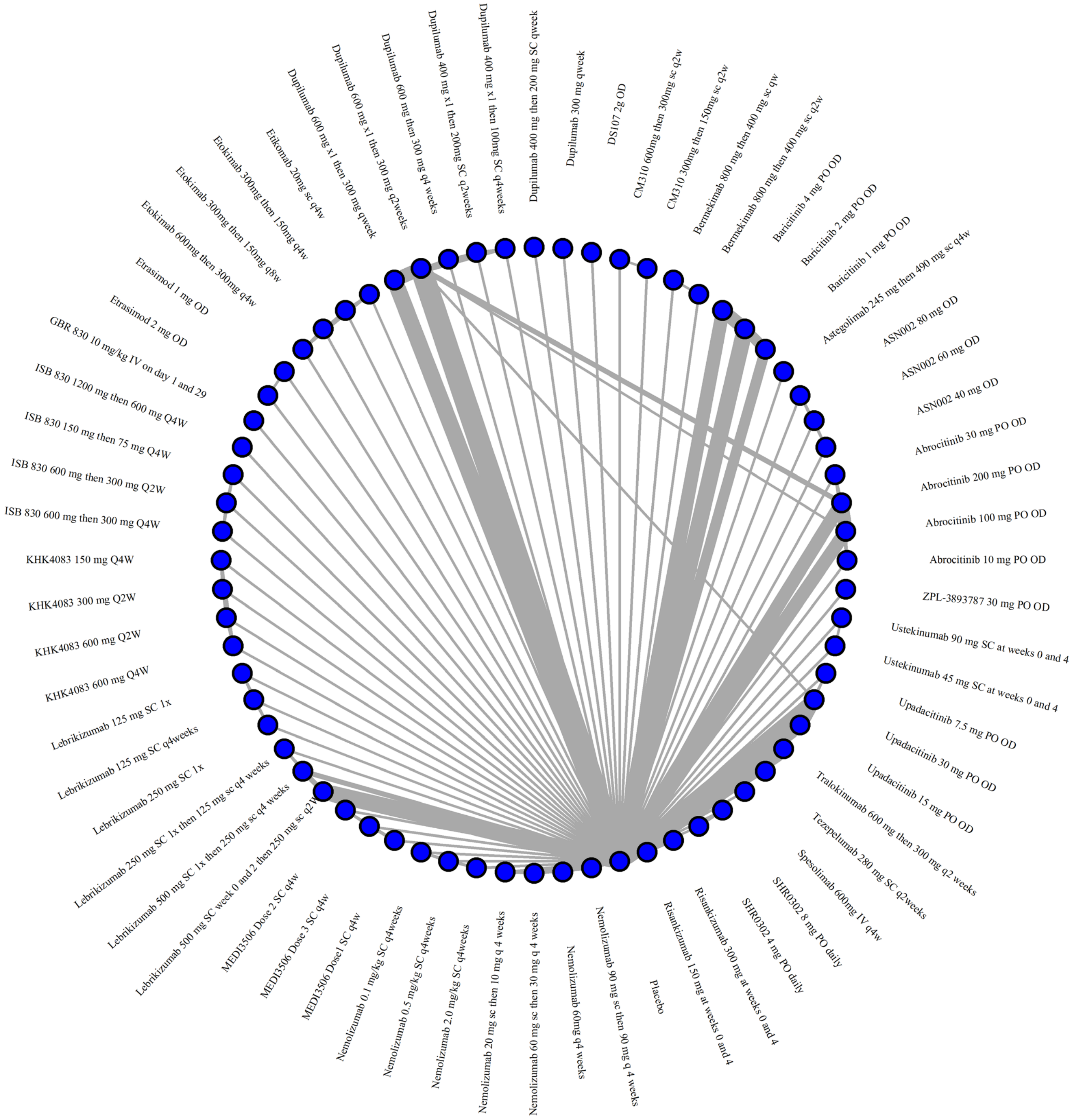

The width of each line connecting two treatments (nodes) is proportional to the number of head-to-head trials for that comparison.
OD: once daily; q1w: once weekly; q2w: every 2 weeks; q4w: every 4 weeks.
*Some studies included in the analyses of trials of adults include a minority proportion of adolescent (12-17 years old) participants.

© 2024 American Medical Association. All rights reserved.

eTable 10. Relative effect estimates for success achieving 75% improvement in EASI (EASI-75) up to 16 weeks of treatment in adults for placebo and medications used in clinical practice. Results for other pairwise comparisons in the network can be requested from the authors. Results are presented as odds ratios (95% credible intervals). Values over 1 indicate greater efficacy for the column-defined treatment. Values less than 1 indicate greater efficacy for the row-defined treatment.

	Abro 100	Abro 200	Bari 2	Bari 4	Dupi	Lebri	Placebo	Tralo	Upa 15	Upa 30
Abro 100		2.1 (1.6, 2.8)	0.6 (0.4, 0.9)	0.7 (0.4, 1)	1.3 (1, 1.8)	1 (0.6, 1.5)	0.2 (0.2, 0.3)	0.5 (0.3, 0.7)	1.7 (1.1, 2.5)	2.8 (1.9, 4.1)
Abro 200	0.5 (0.4, 0.6)		0.3 (0.2, 0.4)	0.3 (0.2, 0.5)	0.6 (0.5, 0.8)	0.5 (0.3, 0.7)	0.1 (0.1, 0.1)	0.2 (0.2, 0.3)	0.8 (0.5, 1.1)	1.3 (0.9, 1.9)
Bari 2	1.8 (1.1, 2.7)	3.7 (2.5, 5.7)		1.2 (0.9, 1.6)	2.3 (1.6, 3.3)	1.7 (1.2, 2.6)	0.4 (0.3, 0.5)	0.9 (0.6, 1.3)	2.9 (2, 4.3)	4.9 (3.4, 7.2)
Bari 4	1.5 (1, 2.4)	3.2 (2.1, 5)	0.9 (0.6, 1.2)		2 (1.4, 2.9)	1.5 (1, 2.3)	0.3 (0.2, 0.5)	0.8 (0.5, 1.1)	2.5 (1.7, 3.8)	4.3 (2.9, 6.4)
Dupi	0.8 (0.5, 1)	1.6 (1.2, 2.1)	0.4 (0.3, 0.6)	0.5 (0.3, 0.7)		0.7 (0.5, 1)	0.2 (0.1, 0.2)	0.4 (0.3, 0.5)	1.2 (0.9, 1.7)	2.1 (1.6, 2.8)
Lebri	1 (0.7, 1.6)	2.2 (1.5, 3.2)	0.6 (0.4, 0.9)	0.7 (0.4, 1)	1.4 (1, 1.9)		0.2 (0.2, 0.3)	0.5 (0.3, 0.7)	1.7 (1.2, 2.5)	2.9 (2, 4.1)
Placebo	4.6 (3.4, 6.4)	9.8 (7.4, 13.3)	2.6 (2, 3.5)	3 (2.2, 4.2)	6.2 (5, 7.5)	4.5 (3.5, 6)		2.3 (1.8, 2.9)	7.7 (5.9, 10)	12.9 (10.2, 16.6)
Tralo	2 (1.4, 3)	4.3 (2.9, 6.4)	1.1 (0.8, 1.7)	1.3 (0.9, 2)	2.7 (2, 3.7)	2 (1.4, 2.9)	0.4 (0.3, 0.6)		3.3 (2.3, 4.8)	5.6 (4, 8.1)
Upa 15	0.6 (0.4, 0.9)	1.3 (0.9, 1.9)	0.3 (0.2, 0.5)	0.4 (0.3, 0.6)	0.8 (0.6, 1.1)	0.6 (0.4, 0.9)	0.1 (0.1, 0.2)	0.3 (0.2, 0.4)		1.7 (1.3, 2.2)
Upa 30	0.4 (0.2, 0.5)	0.8 (0.5, 1.1)	0.2 (0.1, 0.3)	0.2 (0.2, 0.3)	0.5 (0.4, 0.6)	0.4 (0.2, 0.5)	0.1 (0.1, 0.1)	0.2 (0.1, 0.2)	0.6 (0.5, 0.8)	
SUCRA	0.74	0.93	0.53	0.59	0.83	0.73	0.14	0.47	0.89	0.97

Abro 100/200: abrocitinib 100 mg/200 mg daily; bari 2/4: baricitinib 2mg/4 mg daily; dupi: dupilumab 600 mg then 300 mg every 2 weeks; lebri: lebrikizumab 500 mg at week 0 and 2 then 250 mg every 2 weeks; tralo: tralokinumab 600 mg then 300 mg every 2 weeks; upa 15/30: upadacitinib 15mg/30 mg daily.

© 2024 American Medical Association. All rights reserved.

eFigure 7. Network plot for success achieving 90% improvement in EASI (EASI-90) up to 16 weeks of treatment in adults*.

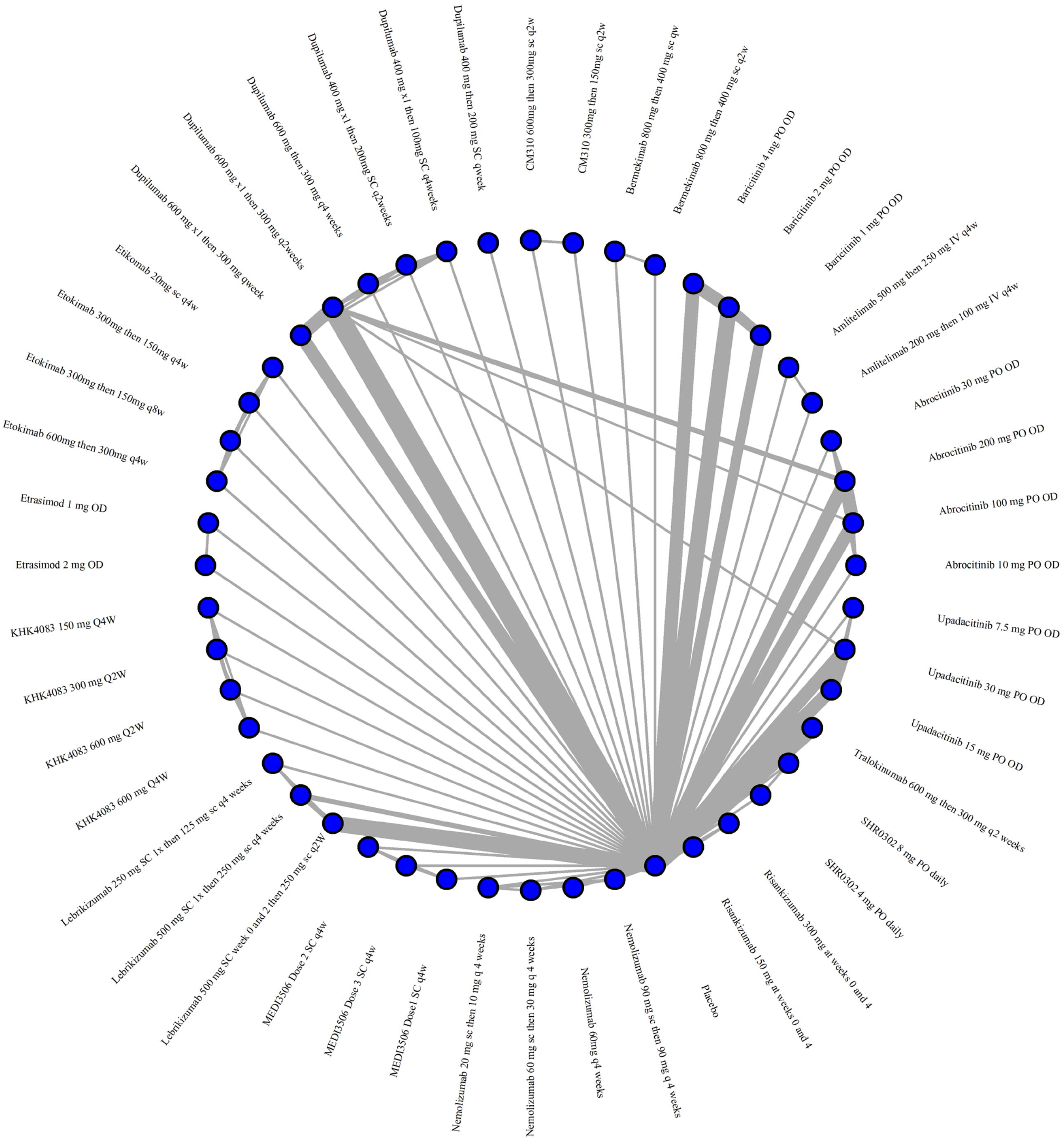

The width of each line connecting two treatments (nodes) is proportional to the number of head-to-head trials for that comparison.
OD: once daily; q1w: once weekly; q2w: every 2 weeks; q4w: every 4 weeks.
*Some studies included in the analyses of trials of adults include a minority proportion of adolescent (12-17 years old) participants.

© 2024 American Medical Association. All rights reserved.

eTable 11. Relative effect estimates for success achieving 90% improvement in EASI (EASI-90) up to 16 weeks of treatment in adults for placebo and medications used in clinical practice. Results for other pairwise comparisons in the network can be requested from the authors. Results are presented as odds ratios (95% credible intervals). Values over 1 indicate greater efficacy for the column-defined treatment. Values less than 1 indicate greater efficacy for the row-defined treatment.

	Abro 100	Abro 200	Bari 2	Bari 4	Dupi	Lebri	Placebo	Tralo	Upa 15	Upa 30
Abro 100		1.9 (1.4, 2.5)	0.4 (0.3, 0.7)	0.6 (0.4, 1)	1.1 (0.8, 1.5)	0.7 (0.5, 1.2)	0.2 (0.1, 0.3)	0.4 (0.3, 0.6)	1.5 (1, 2.3)	2.9 (1.9, 4.3)
Abro 200	0.5 (0.4, 0.7)		0.2 (0.1, 0.4)	0.3 (0.2, 0.5)	0.6 (0.4, 0.8)	0.4 (0.3, 0.6)	0.1 (0.1, 0.1)	0.2 (0.1, 0.3)	0.8 (0.5, 1.2)	1.5 (1.1, 2.2)
Bari 2	2.3 (1.4, 3.7)	4.3 (2.7, 6.8)		1.3 (0.9, 1.9)	2.5 (1.6, 3.8)	1.7 (1, 2.6)	0.4 (0.3, 0.6)	0.9 (0.6, 1.4)	3.5 (2.2, 5.4)	6.6 (4.2, 10.1)
Bari 4	1.7 (1, 2.8)	3.2 (2, 5.2)	0.7 (0.5, 1.1)		1.9 (1.2, 2.9)	1.2 (0.8, 2)	0.3 (0.2, 0.4)	0.7 (0.4, 1.1)	2.6 (1.6, 4.1)	4.9 (3.1, 7.7)
Dupi	0.9 (0.7, 1.2)	1.7 (1.3, 2.2)	0.4 (0.3, 0.6)	0.5 (0.3, 0.8)		0.7 (0.5, 0.9)	0.2 (0.1, 0.2)	0.4 (0.3, 0.5)	1.4 (1, 1.9)	2.6 (2, 3.4)
Lebri	1.4 (0.9, 2.2)	2.6 (1.7, 4)	0.6 (0.4, 1)	0.8 (0.5, 1.3)	1.5 (1.1, 2.2)		0.2 (0.2, 0.3)	0.6 (0.4, 0.8)	2.1 (1.4, 3.1)	4 (2.7, 5.8)
Placebo	5.6 (3.9, 7.9)	10.6 (7.8, 14.3)	2.5 (1.7, 3.5)	3.3 (2.3, 4.8)	6.2 (5, 7.7)	4.1 (3, 5.5)		2.3 (1.7, 3)	8.5 (6.5, 11.2)	16.1 (12.6, 20.6)
Tralo	2.5 (1.6, 3.8)	4.7 (3.1, 6.9)	1.1 (0.7, 1.7)	1.4 (0.9, 2.3)	2.7 (1.9, 3.8)	1.8 (1.2, 2.7)	0.4 (0.3, 0.6)		3.8 (2.6, 5.5)	7.1 (5, 10.2)
Upa 15	0.7 (0.4, 1)	1.2 (0.8, 1.8)	0.3 (0.2, 0.5)	0.4 (0.2, 0.6)	0.7 (0.5, 1)	0.5 (0.3, 0.7)	0.1 (0.1, 0.2)	0.3 (0.2, 0.4)		1.9 (1.5, 2.3)
Upa 30	0.3 (0.2, 0.5)	0.7 (0.5, 0.9)	0.2 (0.1, 0.2)	0.2 (0.1, 0.3)	0.4 (0.3, 0.5)	0.3 (0.2, 0.4)	0.1 (0, 0.1)	0.1 (0.1, 0.2)	0.5 (0.4, 0.7)	
SUCRA	0.67	0.87	0.35	0.47	0.72	0.55	0.09	0.32	0.82	0.93

Abro 100/200: abrocitinib 100 mg/200 mg daily; bari 2/4: baricitinib 2mg/4 mg daily; dupi: dupilumab 600 mg then 300 mg every 2 weeks; lebri: lebrikizumab 500 mg at week 0 and 2 then 250 mg every 2 weeks; tralo: tralokinumab 600 mg then 300 mg every 2 weeks; upa 15/30: upadacitinib 15mg/30 mg daily.

© 2024 American Medical Association. All rights reserved.

eFigure 8. Network plot for achieving success on the Investigator Global Assessment (IGA) scale up to 16 weeks of treatment in adults*.

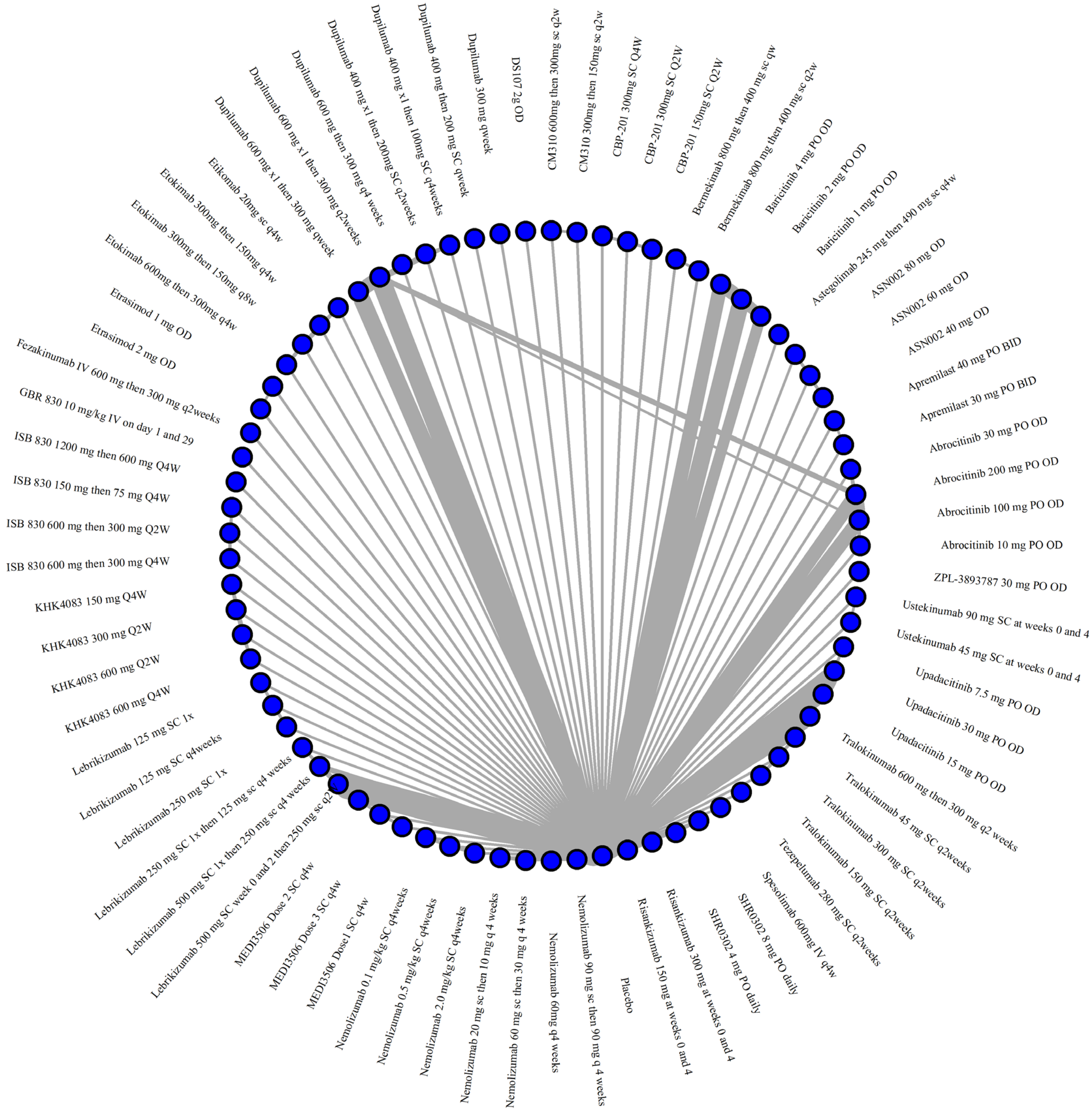

The width of each line connecting two treatments (nodes) is proportional to the number of head-to-head trials for that comparison.

OD: once daily; q1w: once weekly; q2w: every 2 weeks; q4w: every 4 weeks.

*Some studies included in the analyses of trials of adults include a minority proportion of adolescent (12-17 years old) participants.

© 2024 American Medical Association. All rights reserved.

eTable 12. Relative effect estimates for success on the Investigator Global Assessment (IGA) scale up to 16 weeks of treatment in adults for placebo and medications used in clinical practice. Results for other pairwise comparisons in the network can be requested from the authors. Results are presented as odds ratios (95% credible intervals). Values over 1 indicate greater efficacy for the column-defined treatment. Values less than 1 indicate greater efficacy for the row-defined treatment.

	Abro 100	Abro 200	Bari 2	Bari 4	Dupi	Lebri	Placebo	Tralo	Upa 15	Upa 30
Abro 100		1.9 (1.5, 2.5)	0.5 (0.4, 0.9)	0.7 (0.4, 1.1)	1.2 (0.9, 1.6)	0.9 (0.6, 1.4)	0.2 (0.2, 0.3)	0.5 (0.3, 0.7)	1.9 (1.2, 3)	3.5 (2.3, 5.4)
Abro 200	0.5 (0.4, 0.7)		0.3 (0.2, 0.4)	0.4 (0.2, 0.6)	0.6 (0.5, 0.8)	0.5 (0.3, 0.7)	0.1 (0.1, 0.2)	0.2 (0.2, 0.3)	1 (0.7, 1.5)	1.8 (1.2, 2.7)
Bari 2	1.8 (1.2, 2.9)	3.5 (2.3, 5.3)		1.3 (1, 1.8)	2.2 (1.5, 3.2)	1.7 (1.1, 2.5)	0.4 (0.3, 0.6)	0.8 (0.6, 1.2)	3.5 (2.3, 5.3)	6.4 (4.2, 9.7)
Bari 4	1.4 (0.9, 2.2)	2.7 (1.7, 4.2)	0.8 (0.6, 1)		1.7 (1.1, 2.5)	1.3 (0.8, 2)	0.3 (0.2, 0.4)	0.6 (0.4, 1)	2.7 (1.7, 4.2)	4.9 (3.2, 7.6)
Dupi	0.8 (0.6, 1.1)	1.6 (1.2, 2)	0.5 (0.3, 0.7)	0.6 (0.4, 0.9)		0.8 (0.5, 1.1)	0.2 (0.2, 0.2)	0.4 (0.3, 0.5)	1.6 (1.1, 2.3)	2.9 (2, 4.1)
Lebri	1.1 (0.7, 1.7)	2.1 (1.4, 3.1)	0.6 (0.4, 0.9)	0.8 (0.5, 1.2)	1.3 (0.9, 1.9)		0.3 (0.2, 0.3)	0.5 (0.3, 0.7)	2.1 (1.4, 3.1)	3.8 (2.6, 5.7)
Placebo	4.4 (3.2, 6)	8.4 (6.3, 11.2)	2.4 (1.8, 3.3)	3.1 (2.2, 4.4)	5.3 (4.2, 6.6)	4 (3, 5.3)		2 (1.6, 2.5)	8.4 (6.4, 11.1)	15.3 (11.6, 20.3)
Tralo	2.2 (1.5, 3.3)	4.2 (2.9, 6.2)	1.2 (0.8, 1.8)	1.6 (1, 2.4)	2.7 (1.9, 3.7)	2 (1.4, 2.9)	0.5 (0.4, 0.6)		4.3 (2.9, 6.1)	7.8 (5.4, 11.2)
Upa 15	0.5 (0.3, 0.8)	1 (0.7, 1.5)	0.3 (0.2, 0.4)	0.4 (0.2, 0.6)	0.6 (0.4, 0.9)	0.5 (0.3, 0.7)	0.1 (0.1, 0.2)	0.2 (0.2, 0.3)		1.8 (1.5, 2.2)
Upa 30	0.3 (0.2, 0.4)	0.5 (0.4, 0.8)	0.2 (0.1, 0.2)	0.2 (0.1, 0.3)	0.3 (0.2, 0.5)	0.3 (0.2, 0.4)	0.1 (0, 0.1)	0.1 (0.1, 0.2)	0.5 (0.4, 0.7)	
SUCRA	0.72	0.89	0.50	0.61	0.79	0.69	0.17	0.42	0.89	0.96

Abro 100/200: abrocitinib 100 mg/200 mg daily; bari 2/4: baricitinib 2mg/4 mg daily; dupi: dupilumab 600 mg then 300 mg every 2 weeks; lebri: lebrikizumab 500 mg at week 0 and 2 then 250 mg every 2 weeks; tralo: tralokinumab 600 mg then 300 mg every 2 weeks; upa 15/30: upadacitinib 15mg/30 mg daily.

© 2024 American Medical Association. All rights reserved.

eReferences

1. Johnston BC, Thorlund K, Schunemann HJ, et al. Improving the interpretation of quality of life evidence in meta-analyses: the application of minimal important difference units. *Health Qual Life Outcomes*. Oct 11 2010;8:116. doi:10.1186/1477-7525-8-116

2. Schram ME, Spuls PI, Leeflang MM, Lindeboom R, Bos JD, Schmitt J. EASI, (objective) SCORAD and POEM for atopic eczema: responsiveness and minimal clinically important difference. *Allergy*. Jan 2012;67(1):99-106. doi:10.1111/j.1398-9995.2011.02719.x

3. Yosipovitch G, Reaney M, Mastey V, et al. Peak Pruritus Numerical Rating Scale: psychometric validation and responder definition for assessing itch in moderate-to-severe atopic dermatitis. *The British journal of dermatology*. Oct 2019;181(4):761-769. doi:10.1111/bjd.17744

4. Basra MK, Salek MS, Camilleri L, Sturkey R, Finlay AY. Determining the minimal clinically important difference and responsiveness of the Dermatology Life Quality Index (DLQI): further data. *Dermatology*. 2015;230(1):27-33. doi:10.1159/000365390

5. Santesso N, Glenton C, Dahm P, et al. GRADE guidelines 26: informative statements to communicate the findings of systematic reviews of interventions. *Journal of clinical epidemiology*. Mar 2020;119:126-135. doi:10.1016/j.jclinepi.2019.10.014

6. Hutton B, Salanti G, Caldwell DM, et al. The PRISMA extension statement for reporting of systematic reviews incorporating network meta-analyses of health care interventions: checklist and explanations. *Ann Intern Med*. Jun 02 2015;162(11):777-84. doi:10.7326/M14-2385

© 2024 American Medical Association. All rights reserved.
