# Supplementary material for: Systemic Immunomodulatory Treatments for Atopic Dermatitis: Living Systematic Review and Network Meta-Analysis Update
Source: JAMA Dermatol. 2024 Jul 17;160(9):936–44. doi: 10.1001/jamadermatol.2024.2192 (PMC11255974; doi:10.1001/jamadermatol.2024.2192)
Supplement: Supplement 3. — Data Sharing Statement [file jamadermatol-e242192-s003.pdf]

Data Sharing Statement

Drucker. Systemic Immunomodulatory Treatments for Atopic Dermatitis. *JAMA Dermatol*. Published July 17, 2024. doi:10.1001/jamadermatol.2024.2192

Data
Data available: Yes
Data types: Data (not involving human participants)
How to access data: We will submit an excel file with the manuscript that can be uploaded as supplemental material. The Excel file will also be available for download from wwww.eczematherapies.com/research
When available: With publication

Supporting Documents
Document types: Statistical/analytic code
How to access documents: Statistical code for the living NMA is available at www.eczematherapies.com/research.
When available: beginning date: 02-27-2024

Additional Information
Who can access the data: Publicly available
Types of analyses: Any purpose
Mechanisms of data availability: Available for download
Any additional restrictions: None
